# Effects of neuregulin-1 administration on neurogenesis in the adult mouse hippocampus, and characterization of immature neurons along the septotemporal axis

**DOI:** 10.1038/srep30467

**Published:** 2016-07-29

**Authors:** Ian Mahar, Angus MacIsaac, John Junghan Kim, Calvin Qiang, Maria Antonietta Davoli, Gustavo Turecki, Naguib Mechawar

**Affiliations:** 1 McGill Group for Suicide Studies, Douglas Mental Health University Institute, 6875 LaSalle Blvd, Verdun, Québec, H4H 1R3, Canada; 2 Integrated Program in Neuroscience, McGill University, Montreal Neurological Institute, 3801 University Street, Montréal, QC H3A 2B4, Canada; 3 Department of Psychiatry, McGill University, 1033 Pine Avenue West, Montréal, QC H3A 1A1, Canada.

## Abstract

Adult hippocampal neurogenesis is associated with learning and affective behavioural regulation. Its diverse functionality is segregated along the septotemporal axis from the dorsal to ventral hippocampus. However, features distinguishing immature neurons in these regions have yet to be characterized. Additionally, although we have shown that administration of the neurotrophic factor neuregulin-1 (NRG1) selectively increases proliferation and overall neurogenesis in the mouse ventral dentate gyrus (DG), likely through ErbB3, NRG1’s effects on intermediate neurogenic stages in immature neurons are unknown. We examined whether NRG1 administration increases DG ErbB3 phosphorylation. We labeled adultborn cells using BrdU, then administered NRG1 to examine *in vivo* neurogenic effects on immature neurons with respect to cell survival, morphology, and synaptogenesis. We also characterized features of immature neurons along the septotemporal axis. We found that neurogenic effects of NRG1 are temporally and subregionally specific to proliferation in the ventral DG. Particular morphological features differentiate immature neurons in the dorsal and ventral DG, and cytogenesis differed between these regions. Finally, we identified synaptic heterogeneity surrounding the granule cell layer. These results indicate neurogenic involvement of NRG1-induced antidepressant-like behaviour is particularly associated with increased ventral DG cell proliferation, and identify novel distinctions between dorsal and ventral hippocampal neurogenic development.

Adult hippocampal neurogenesis is the process by which new neurons are added throughout life to the granule cell layer of the dentate gyrus (DG) in the hippocampus^1^. This phenomenon has been associated with a number of different functions, most notably learning and memory, affective regulation, and response to stress^2^,^3^. In addition, impaired neurogenesis and reduced number of granule cells have been associated with depression^4^,^5^, whereas antidepressant treatment increases hippocampal neurogenesis in rodents, non-human primates, and humans^6^,^7^,^8^. The functional role of new granule cell neurons appears to be related to their unique electrophysiological properties during a hyperplastic window extending from approximately four to six weeks of cellular age in rodents^9^, whereas mature granule cells are comparatively quiescent^10^.

Anatomically, the hippocampus follows a septotemporal axis, progressing from the dorsal (or septal) hippocampus rostrally to the ventral (or temporal) hippocampus caudally^11^,^12^,^13^. Recent studies have established that the dorsal and ventral hippocampus are anatomically and functionally distinct regions, with the dorsal hippocampus (analogous to the posterior hippocampus in humans) being more involved in spatial learning and memory, whereas the ventral hippocampus (anterior hippocampus in humans) is associated more with emotional regulation^11^,^14^,^15^. This functional distinction is maintained at the level of the DG and DG neurogenesis, as studies have shown that dorsal hippocampal neurogenesis is necessary for contextual discrimination, whereas the ventral dentate gyrus has been associated with affective behaviour, antidepressant response, and response to stress^12^,^13^,^16^,^17^,^18^. As a result, it has been proposed that the influence of adult hippocampal neurogenesis on emotion-related behavior is contingent upon the number of functional immature hyperplastic neurons present in the ventral DG specifically at the time of behavioral assessment, potentially due to connections between the ventral hippocampus and the hypothalamic-pituitary-adrenal (HPA) axis, amygdala, nucleus accumbens, and prefrontal cortex^19^. However, despite the identified importance of these cells in emotion-related behavior, there is a lack of studies specifically examining developmental, morphological, and synaptic features distinguishing immature ventral and dorsal hippocampal neurons.

In addition to the influence of monoaminergic agents, such as antidepressant medications, on adult hippocampal neurogenesis, neurotrophic factors have also been shown to increase hippocampal neurogenesis^19^. These include brain-derived neurotrophic factor (BDNF)^20^,^21^, vascular endothelial growth factor (VEGF)^22^, insulin-like growth factor (IGF)^23^, and most recently neuregulin-1 (NRG1)^12^. NRG1, which had previously been associated with affective behaviour and psychopathology including schizophrenia, bipolar disorder, and depression in multiple studies^24^,^25^,^26^,^27^,^28^, was found in this study to rapidly increase hippocampal proliferation and neurogenesis selectively in the caudal dentate gyrus within the ventral hippocampus when administered peripherally to young adult mice^12^. This neurogenic effect of subchronic NRG1β administration likely resulted from local binding to NRG1 receptor ErbB3 in neural progenitor cells (whereas ErbB4, the other primary NRG1 receptor that the ligand binds to directly, was not observed in early neurogenic cells), and was associated with antidepressant-like behaviour^12^. However, although these results identified a temporal association between increased proliferation/neurogenesis and antidepressant-like behavior, the effects of NRG1 on intermediate stages of hippocampal neurogenesis, occurring between cell proliferation and functionality several weeks later, remain to be assessed. In addition, activation of DG ErbB3 by NRG1 administration has not been demonstrated.

In the present study, we examined the consequences of NRG1 administration on immature neurons in the young adult hippocampus, to determine if the mood-related behavioral effects of NRG1 administration previously associated with increased hippocampal neurogenesis can be specifically identified as being due to changes to proliferation/neurogenesis, or alternatively whether other morphological and synaptic changes to neurons in intermediate stages of development may be involved in this phenomenon. This was accomplished through assessments of neuronal survival, morphometric analyses of reconstructed immature neurons, and indirect quantifications of synaptic densities through PSD-95-immunoreactive puncta. In this way, our experimental design was similar to our previous study^12^; however, in the current study, we focused on distinct neurogenic stages, specifically examining immature neurons at an age between neuronal differentiation and maturity, in order to further characterize the effects of NRG1 on hippocampal neurogenesis across neurogenic stages. We also confirmed that NRG1 administration activates ErbB3 selectively in the ventral DG, and this activation did not result in neurogenic changes in immature neurons, in contrast to proliferating cells as shown previously^12^. In addition, we applied these data to characterize immature neurons in the dorsal and ventral DG, in order to identify both common and distinguishing features between these cell populations contributing to vastly distinct behaviorally- and psychopathologically-relevant functions.

Our findings reveal that the effects of NRG1 on hippocampal neurogenesis are temporally and subregionally specific (i.e. pertaining only to proliferation in the ventral DG), and lead to increased neurogenesis selectively in the ventral DG. Furthermore, our results identify novel differences between immature neurons in the dorsal and ventral DG, with respect to specific developmental features that may contribute to their specific functional contributions.

## Experimental Procedures

### Animals

Young adult male C57Bl/6 mice were obtained from Charles River (Quebec), and were approximately two months of age at the time of experimentation. Mice were housed on a 12:12 light:dark cycle, with *ad libitum* access to food and water. All experiments adhered to the policies and guidelines of the Canadian Council on Animal Care, and were approved by McGill University’s Animal Care Committee.

### NRG1β and BrdU administration

For phosphorylated ErbB3 (pErbB3) quantification, mice were administered either sterile 0.9% saline or NRG1β type-I (R&D Systems; EGF domain dissolved in sterile 0.9% saline, administered at a constant rate of 10 μg/d; n = 8/group) dissolved in saline for 24 h through subcutaneously-implanted osmotic mini-pumps (Alzet), as performed and described previously^12^. Post-operatively, mice were placed on a heating pad to recover, monitored for complications, and received an anti-inflammatory Carprofen tablet placed in the cage.

To examine neurogenic stages and immature neurons, BrdU (50 mg/kg) was injected i.p. three times, spaced ≥3 hours apart within a 24 h period in order to label dividing cells. 15 days after BrdU administration, mice were given either recombinant NRG1 or vehicle (n = 10) for three days in subcutaneously-implanted osmotic mini-pumps. This latency, in contrast to the delays in our previous studies^12^ was chosen as it corresponds to a specific critical period during which adultborn neurons undergo a period of apoptosis or survival, extend dendrites into the molecular layer, and form synapses, all as part of immature neuronal development^29^,^30^,^31^,^32^ (Fig. S1). Mice were left to recover post-operatively on a heating pad, and each animal was given an anti-inflammatory Carprofen tablet.

### Tissue processing

For pErbB3 quantification, following NRG1 or saline administration mice were deeply anesthetized with isoflurane and decapitated. Brains were removed and placed in cold PBS under a dissecting microscope. DGs were dissected based on a previously established DG isolation procedure^33^. Dissected DGs were separated into dorsal and ventral segments, corresponding to previously established designations^12^, and stored at −80 °C. Protein was digested in RIPA buffer containing protease and phosphatase inhibitors, then sonicated and centrifuged at 13200 RPM for 20 min at 4 °C. Supernatant was removed, vortexed, aliquoted, and stored at −20 °C, and protein verified with a BSA protein assay kit (Pierce) and plate reader at 562 nm.

For immature neuronal experiments, mice were deeply anesthetized with a ketamine, xylazine and acepromazine cocktail (0.1 ml/100 g), and perfused intracardially with ice-cold phosphate-buffered saline (PBS), then with 4% formaldehyde in 0.1 M phosphate buffer. Brains were rapidly removed, postfixed at 4 °C for 24 h in the fixative solution, then transferred to 30% sucrose in PBS until equilibrium was reached. Brains were flash frozen to −40 °C in isopentane and cut with a cryostat into serial 40 μm coronal sections that were immersed in a cryoprotectant solution (glycerol:ethylene glycol:PBS, 3:3:4) and stored at −20 °C.

### Fluorescence-Assisted Cell Sorting

To validate DG dissections, parallel DG dissections were performed in adult transgenic mice expressing GFP at the nestin promoter (nestin-GFP^12^,^34^) on a C57Bl/6 background and non-transgenic littermates, mimicking the pErbB3 NRG1/saline administration experiments. DG tissue was digested, triturated, and processed following an established cell isolation procedure^35^, and neuronal gradient fraction cells were sorted on a FACSAria machine (BD Biosciences). Fluorescence phenotype of sorted cells was verified on an Olympus BX51 microscope.

### pErbB3 quantification

ErbB3 phosphorylation was assessed using a phospho-ErbB3-specific ELISA kit (RayBiotech). Briefly, dorsal and ventral samples from subjects were loaded onto wells of the kit’s 96-well plate, with subsequent manufacturers’ instructions observed. Samples were read on a SpectraMax plate reader (Molecular Devices) at 450 nm. To verify and correct for protein concentration, samples were loaded onto 4–12% polyacrylamide gels (Invitrogen), transferred to nitrocellulose membrane (Amersham GE Healthcare), treated with Ponceau (Sigma) and visualized using a charge-coupled device camera and Image Studio Lite software (Licor). Values were normalized to control samples.

### Immunohistochemistry

Immunohostochemistry (IHC) was conducted on free-floating sections. For each protocol, distinct subseries of sections were washed with PBS (pH = 7.2) between incubations, except between blocking and primary incubation. All steps occurred at room temperature (RT) except for incubation in HCl/PBS, which was at 37 °C, and overnight primary incubations, which were at 4 °C. Steps following addition of fluorescent antibodies included protection from light.

For BrdU single-labeling IHC to assess cell survival and cytogenesis, sections were incubated for 1.5 h in PBS containing 0.2% Triton X-100 (Fisher; PBS-T), then 10 min in 0.9% H_2_O_2_ in PBS, followed by 30 min in 2 N HCl in PBS, 30 min in block solution containing PBS-T with 2% normal goat serum (Vector), and then overnight in block solution containing rat anti-BrdU antibody (1:1000; Serotec). This was followed by incubation in biotinylated goat anti-rat secondary antibody (1:200; Vector) in block solution for 1 h, and the labeling revealed with DAB using VectaStain Elite and DAB kits (both by Vector), and quenching in ddH2O for 5 min before transferring sections to PBS. Sections were mounted on SuperFrost Plus slides (Fisher) in PBS-T, left to dry to affix to slides, then dehydrated using increasing concentrations of ethanol followed by xylene, and coverslipped using Permount (Fisher).

For BrdU/doublecortin (DCX) double-labeling IHC for neuronal reconstructions, we used a combination of light and fluorescence microscopy to optimize cell identification while avoiding issues of photobleaching during tracing. Sections were incubated in PBS-T for 2 h, then 10 min in 0.9% H_2_O_2_ in PBS, followed by 1 h in block solution containing PBS-T with 2% normal horse serum (Vector), then 24 h in block solution containing goat anti-DCX primary antibody (1:250; Santa Cruz). Sections were then incubated for 90 min in block solution containing biotinylated horse anti-goat secondary antibody (1:200), then VectaStain Elite and DAB as performed with BrdU IHC. We then incubated sections for 30 min in 1 N HCl in PBS, then 1 h in block solution comprised of PBS-T with 2% normal goat serum, then anti-BrdU in block solution overnight. Goat anti-rat DyLight 594 (1:500; Jackson) secondary antibody in block solution was added for 90 min. Sections were mounted in PBS-Ton to slides, then rinsed in ddH2O and coverslipped with ProLong Gold antifade reagent with DAPI (Life Technologies) using #1.5 thickness cover slips (Corning).

For BrdU/DCX/PSD-95 triple-labeling (Fig. S2) as an estimate of synaptic density, sections were treated for 1 h with PBS-T, then 1 h in blocking solution (PBS-T containing 2% normal donkey serum (Jackson)) followed by overnight incubation in blocking solution containing anti-DCX and rabbit anti-PSD-95 (1:250; Invitrogen) antibodies. Sections were then incubated in blocking solution containing donkey anti-goat DyLight 488 and donkey anti-rabbit DyLight 647 secondary antibodies (1:500; Jackson), followed by 1 N HCl in PBS for 30 min, then 1 h block in PBS-T containing 2% normal goat serum, followed by overnight incubation in blocking solution containing anti-BrdU, then 75 min in PBS-T containing goat anti-rat DyLight 594 secondary antibody (1:500; Jackson). Sections were mounted on slides, rinsed in ddH2O, and coverslipped with ProLong Gold antifade reagent with DAPI using #1.5 thickness cover slips. A similar protocol was used for staining for confocal microscopy in order to verify PSD-95 staining specificity. Confocal imaging of BrdU/DCX colocalization using a similar protocol is also available in a previous publication^12^.

### Cell quantification

BrdU-immunoreactive (-IR) cells were counted across the septotemporal axis on a Leica CME microscope, using a 40× 0.65 NA E2 achromat objective. Each labeled neurogenic cell was counted in the DG, as previous studies have suggested that this may be preferable to estimates derived from stereological methods for neurogenic studies due to cell distribution heterogeneity^36^. For this and other experiments discussed herein, experimenters were blind to group identity. Dorsal and ventral DG regions were identified using previously established criteria^12^,^37^,^38^,^39^; dorsal: −1.46 to −2.54 mm from bregma; ventral: −2.55 to −3.80 mm from bregma). The serial section fraction (SSF) was 1/8. Data are expressed as the average number of BrdU-IR cells per section.

### Neuronal reconstruction

At least six clearly-labeled BrdU-/DCX-IR immature neurons per subject across the septotemporal axis from multiple DGs were randomly selected to be reconstructed using Neurolucida software (MBF). Neurons were traced using an Olympus BX51 microscope with a motorized stage and CX-9000 camera using a 100X (1.40 NA UPlansApo oil objective), and analyzed using Neurolucida Explorer (MBF Bioscience). The microscope was calibrated for grid tuning and parcentric/parfocal parameters. Labeled cells with primary or secondary dendrites which appeared to be cut off by the upper or lower boundaries of the tissue, or without a visible process extending through the granule cell layer, were excluded from analysis. Cell bodies were traced in their primary plane of focus, and analysed with the other neuronal reconstruction parameters. The image showing both DCX DAB IHC and BrdU immunofluorescence was produced in Adobe Illustrator by overlaying a fluorescence (50% transparency) image over a brightfield image of the same field.

### Assessment of synapse-related immunoreactive puncta

Synaptic density was assessed indirectly by quantification of synapse-related immunoreactivity in two stages, with the first examining overall synaptic density (using PSD-95 expression as an approximation) within the molecular layer surrounding the granule cell layer, and the second examining synapse-related protein expression specifically associated with ~18 day old immature BrdU-IR neurons. These experiments used an Axio Imager.M2 microscope with motorized stage, AxioCam MR camera, and Apotome.2 system (Zeiss), at 63× (1.40 NA plan-apochromat oil objective), which allowed fine resolution of fluorescent signals. The microscope was calibrated for grid tuning and parcentric/parfocal parameters.

To estimate overall synaptic density, four zones in the molecular layer were chosen: adjacent to the basal aspect of the suprapyramidal blade, adjacent to the apical suprapyramidal blade, adjacent to the basal infrapyramidal blade, and adjacent to the apical infrapyramidal blade. To clarify, the suprapyramidal blade is the more dorsal in the dorsal DG, and more lateral in the ventral DG, with the converse being true for the infrapyramidal blade, and the apical aspect is nearest the confluence of the two blades. For each zone, a z-stack of images from the top to the bottom of the tissue was obtained using Neurolucida software. Four DGs (two dorsal, two ventral) were included per subject. Image acquisition parameters were maintained between subjects. For each image stack, the intensity of PSD-95 staining was obtained from luminance information in each slice for which the molecular layer was in focus without tissue aberration, then averaged between slices. Additional analyses using all images in each stack throughout the issue did not change statistical significance status of primary comparisons.

To estimate synapse-related immunoreactive puncta numbers and density specifically for immature neurons, we traced BrdU-/DCX-IR cells at 63X using three-dimensional virtual tissue images from NeuroLucida software. We then traced the neuron cell body and dendrite along the three dimensions, and counted PSD-95-IR puncta contacting cellular dendrites or cell soma across dendritic branch orders and on the soma for 122 cells across all subjects and the septotemporal axis. Triple-labeled cells were identified using widefield fluorescence microscopy, and quantifications were obtained using the Apotome system visualizing DCX/PSD-95 IR in these cells. For densitometric analyses, dendritic counts were relative to dendritic length, and somatic counts were relative to somatic area.

### Confocal microscopy

PSD-95 staining specificity and reliability were assessed with a Zeiss LSM510 Meta confocal microscope equipped with an Axiovert 200 M stand and motorized stage, and 488 nm, 543 nm, and 633 nm wavelength lasers (Carl Zeiss Canada). Objectives used were 40X Plan-Neofluar 1.3 NA oil and 100X Plan-Apochromat 1.4 NA oil. Images were obtained using the Zeiss Aim software package (Carl Zeiss Canada), with a pixel dwell time of ≥3.20 μs, optical slice 1–3 μm sampled at 1 μm intervals. PSD-95 was absent in control tissue lacking treatment with primary antibody. PSD-95 staining was absent in corpus callosum but present in synapse-rich regions such as cortical and hippocampal regions (Fig. S3), suggesting specificity in our PSD-95 labeling.

### Statistics

Pairwise comparisons were made using paired or unpaired t-tests with or without Welch’s correction for parametric data, or Mann-Whitney or Wilcoxon tests for non-parametric data. Region x treatment analyses were conducted using two-way mixed-model ANOVAs, with Holm-Sidak multiple comparisons post-hoc tests; treatment was a between-subjects factor, and DG subregion was a within-subjects factor. Cytogenic relationships were analyzed by Pearson correlations. Tests between NRG1- and saline-treated animals were between-subjects designs, whereas analyses between dorsal and ventral DG parameters in the same animals were within-subjects designs. All statistical tests were two-tailed, and p values ≤ 0.05 were considered statistically significant. Figures show mean ± SEM.

## Results

### Peripheral NRG1 phosphorylates ErbB3 in the ventral DG

**Figure 1:**
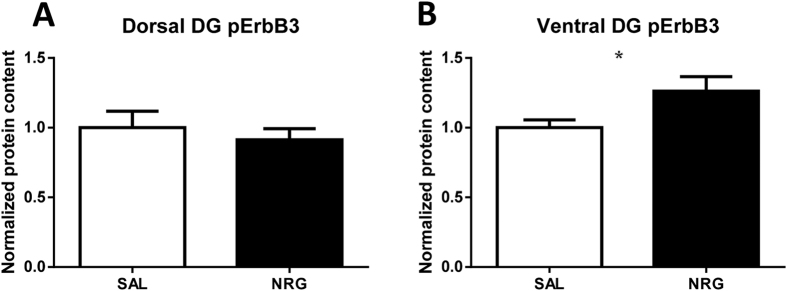
Quantification of phosphorylated ErbB3. Ventral (**A**) (but not dorsal (**B**)) dentate gyrus (DG) ErbB3 phosphorylation is increased by 24 h of subcutaneous NRG1. *p < 0.05. FC, fold change; NRG, neuregulin; SAL, saline.

After 24 hours of NRG1 administration, ErbB3 phosphorylation was selectively increased in the ventral DG (p = 0.044; Fig. 1A,B), in agreement with previous findings showing that this administration paradigm selectively increases cell proliferation in this subregion^12^. FACS in nestin-GFP transgenic animals revealed a GFP+ population in DG dissections (Fig. S4), supporting the anatomical accuracy of DG dissections.

### Effects of neuregulin-1 administration on cell survival

**Figure 2:**
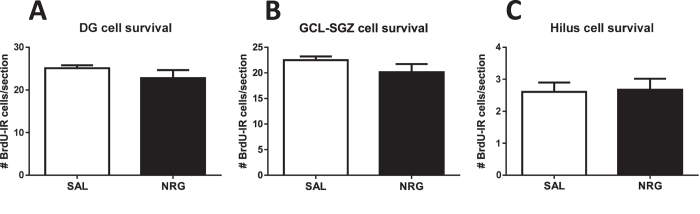
Effects of neuregulin-1 administration on cell survival. Cell survival is not affected by NRG1 administration in the overall DG (**A**), or specifically in the granule cell layer and SGZ (**B**) or hilus (**C**). SAL, saline.

Summarized effects of NRG1 administration across neurogenic stages from current and previous experiments^12^ are available in Table 1. In mice administered NRG1 starting 15 days after BrdU injections, overall DG cell survival, as assessed by average numbers of BrdU-IR cells per section, was unaffected by NRG1 treatment. This was true for the overall number of BrdU-IR cells in the DG (p = 0.27; Fig. 2A), for the subgranular zone and granule cell layer (SGZ-GCL) alone (p = 0.21; Fig. 2B), and for the hilus alone (p = 0.89; Fig. 2C). In examining the effect of NRG1 on hippocampal subregions, there was also no difference specifically in the dorsal DG (total: p = 0.19; hilus: p = 0.67; SGZ/GCL: p = 0.21; Fig. S5A–C) or in the ventral DG (total: p = 0.44; hilus: p = 0.67; SGZ/GCL: p = 0.30; Fig. S5D–F).

### Effects of neuregulin-1 administration on morphological development

**Figure 3:**
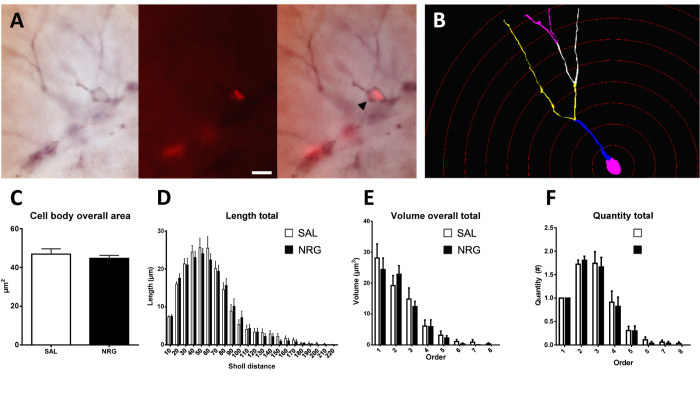
Effects of neuregulin-1 administration on dendritic morphological development. (**A**) Immature neuron (arrowheads) labeled with DCX (left) and BrdU (middle) in the same field, with an overlay at right. (**B**) example reconstructed neuron showing dendritic branch orders and Sholl distances (10 μm). NRG1 administration did not affect immature neuron morphology, including cell body size (**C**), dendritic length (**D**), dendritic volume (**E**), and number of dendritic branches (**F**). Scale bar = 10 μm. SAL, saline.

BrdU-/DCX-IR (Fig. 3A,B) cell body perimeter did not differ significantly between NRG1- and saline-treated mice overall (averaged across the septotemporal axis; p = 0.36; Fig. S6A), or in the dorsal (p = 0.07; Fig. S6B) or ventral (p = 0.54; Fig. S6C) subregions. This was also the case for cell body area (overall: p = 0.49; dorsal: p = 0.13; ventral: p = 0.54; Fig. 3C, S5AB–AC), feret max (p = 0.59; p = 0.13; p = 0.54; Fig. S6D–F), feret min (p = 0.60; p = 0.39; p = 0.83; Fig. S6G–I), aspect ratio (p = 0.95; p = 0.20; p = 0.83; Fig. S6J–L), compactness (p = 0.98; p = 0.46; p = 0.82; Fig. S6M–O), convexity (p = 0.098; p = 0.16; p = 0.61; Fig. S6P–R), form factor (p = 0.21; p = 0.14; p = 0.99; Fig. S6S–U), roundness (p = 0.92; p = 0.49; p = 0.88; Fig. S6V–X), and solidity (p = 0.39; p = 0.31; p = 0.62; Fig. S6Y–AA).

Total dendritic length did not differ with treatment overall (p = 0.81; Fig. S7A), or in the dorsal (p = 0.86; Fig. S7B) or ventral (p = 0.70; Fig. S7C) subregions. Examining length by branch order, there was no effect of treatment overall (p = 0.82; Fig. S8A), or in the dorsal (p = 0.96; Fig. S8B) or ventral (p = 0.60; Fig. S8C) subregions, and this was also the case for Sholl analysis of length (overall: p = 0.81; dorsal: p = 0.98; ventral: p = 0.60; Fig. 3D, S7W–X). Mean dendritic length (length per branch) by branch order did not differ with NRG1 treatment overall (p = 0.91; Fig. S8D), or in the dorsal (p = 0.74; Fig. S8E) or ventral (p = 0.38; Fig. S8F) subregions. There was a significant effect for branch order overall and in both subregions (ps < 0.0001); length was highest in the 2^nd^ and 3^rd^ branch orders versus subsequent branches (ps ≤ 0.0021), and these tended to be higher than the 1^st^ order (ps ≤ 0.089).

Total dendritic volume did not differ with treatment overall (p = 0.69; Fig. S7D), or in the dorsal (p = 0.81; Fig. S7E) or ventral (p = 0.65; Fig. S7F) subregions. Examining volume by branch order, there was no effect of treatment overall (p = 0.70; Fig. 3E), or in the dorsal (p = 0.99; Fig. S8S) or ventral (p = 0.56; Fig. S8T) subregions. Mean dendritic volume by branch order did not differ with NRG1 treatment overall (p = 0.67; Fig. S8G), or in the dorsal (p = 0.91; Fig. S8H) or ventral (p = 0.46; Fig. S8I) subregions.

Total dendritic surface area did not differ with treatment overall (p = 0.66; Fig. S7G), or in the dorsal (p > 0.99; Fig. S7H) or ventral (p = 0.66; Fig. S7I) subregions. Examining surface area by branch order, there was no effect of treatment overall (p = 0.68; Fig. S8J), or in the dorsal (p = 0.89; Fig. S8K) or ventral (p = 0.54; Fig. S8L) subregions. Mean dendritic surface area by branch order did not differ with NRG1 treatment overall (p = 0.64; Fig. S8M), or in the dorsal (p = 0.98; Fig. S8N) or ventral (p = 0.38; Fig. S8O) subregions.

Total dendritic nodes did not differ with treatment overall (p = 0.84; Fig. S7J), or in the dorsal (p = 0.37; Fig. S7K) or ventral (p = 0.61; Fig. S7L) subregions. Examining nodes by branch order, there was no effect of treatment overall (p = 0.84; Fig. S8P), or in the dorsal (p = 0.59; Fig. S8Q) or ventral (p = 0.82; Fig. S8R) subregions, and this was also the case for Sholl analysis of nodes (overall: p = 0.84; dorsal: p = 0.56; ventral: p = 0.82; Fig. S8Y–AA). There was a significant effect for Sholl distance overall and in both subregions (ps < 0.0001). Overall nodes were most prevalent 10–70 μm from the cell body (ps ≤ 0.0004, except 60–70 versus 70–80), in the dorsal DG nodes were most prevalent 10–60 um from cell body (ps ≤ 0.0048, except versus 60–70 um and 40–50 versus 70–80), and ventrally nodes were most prevalent 10–60 um from cell body (ps ≤ 0.0024, except versus 60–70 um).

Quantity by branch order did not differ with NRG1 treatment overall (p = 0.79; Fig. 3F), or in the dorsal (p = 0.55; Fig. S8U) or ventral (p = 0.88; Fig. S8V) subregions.

Total dendritic endings did not differ with treatment overall (p = 0.73; Fig. S7M), or in the dorsal (p = 0.33; Fig. S7N) or ventral (p = 0.73; Fig. S7O) subregions, and this was also the case for Sholl analysis of endings (overall: p = 0.73; dorsal: p = 0.49; ventral: p = 0.83; Fig. S8AB–AD).

Dendritic intersections by Sholl analysis did not differ with NRG1 treatment overall (p = 0.66; Fig. S8AE), or in the dorsal (p = 0.85; Fig. S8AF) or ventral (p = 0.56; Fig. S8AG) subregions.

Total dendritic tortuosity did not differ between NRG1- and saline- treated mice overall (p = 0.68; Fig. S7P), or in the dorsal (p = 0.70; Fig. S7Q) or ventral (p = 0.85; Fig. S7R) subregions.

### Effects of neuregulin-1 administration on synapse development

**Figure 4:**
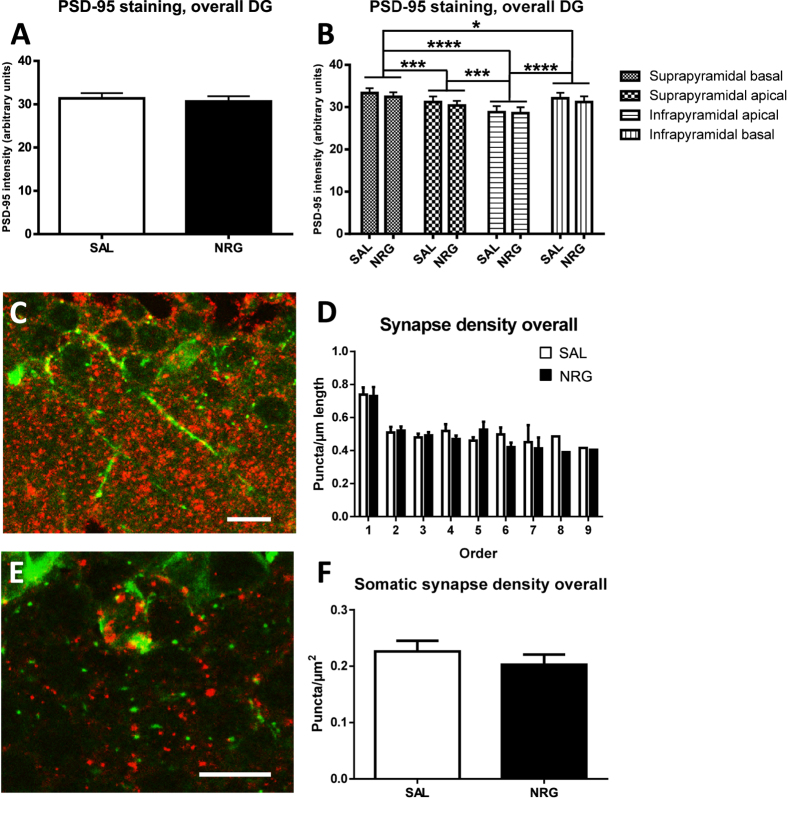
Effects of neuregulin-1 administration on synapse development. Overall synaptic density (approximated using PSD-95) in the molecular layer surrounding the DG was not affected by NRG1 administration (**A**), and this was also the case in analyzing particular zones adjacent to various aspects of the DG (**B**), although there was heterogeneity in synapse-related immunoreactivity between these zones. (**C**) dendritic synapse-related labeling by DCX (green) and PSD-95 (red) staining in BrdU-identified immature neurons. (**D**) Dendritic synapse-related puncta density was unaffected by NRG1 administration. (**E**) somatic synaptic labeling by DCX (green) and PSD-95 (red) staining in BrdU-identified immature neurons. Somatic synapse-related puncta density was unaffected by NRG1 administration (**F**). Scale bar = 10 μm. **p ≤ 0.01; ****p ≤ 0.0001. SAL, saline.

NRG1 treatment did not affect overall molecular layer synaptic density (as approximated using PSD-95 expression) averaged across zones (p = 0.68; Fig. 4A). This was consistent along the dorsal (p = 0.42; Fig. S9A) and ventral (p = 0.17; Fig. S9B) subregions. When analyzing individual zones, there was no significant effect of treatment overall (p = 0.68; Fig. 4B) or in the dorsal (p = 0.42; Fig. S9C) or ventral (p = 0.17; Fig. S9D) subregions.

Examining synapse-related immunoreactivity specifically for immature neurons, treatment with NRG1 did not affect overall dendritic synapse-related puncta numbers (p = 0.43; Fig. S9E) or density (p = 0.82; Fig. S9H). This was also the case for the dorsal (total synapses: p = 0.82, Fig. S9F; density: p = 0.48; Fig. S9I) and ventral (total synapses: p = 0.53, Fig. S9G; density: p = 0.19; Fig. S9J) subregions.

For branch order synapse-related analyses, we analyzed the first four branch orders, as all subjects had cells with dendrites reaching at least this order (barring one subject with fewer dorsal branch orders). Analyzing by overall branch order, NRG1 treatment did not affect number (p = 0.43) or density (p = 0.82) of dendritic synapse-related immunoreactivity (Fig. 4C,D, S9K), although there were significant effects of branch order (ps < 0.0001), with lower numbers of puncta in the first two branches than subsequent two branches (ps ≤ 0.0020) and lower numbers in 1^st^ versus 2^nd^ order branches (p = 0.0019), and highest density in the 1^st^ branch order (ps < 0.0001). In the dorsal DG, NRG1 did not affect number (p = 0.86; Fig. S9L) or density (p = 0.75; Fig. S9N) of synapse-related puncta, although there were significant effects of branch order (ps < 0.0001), with lower numbers of puncta in the 1^st^ branch than subsequent three branches (ps ≤ 0.01), and highest density in the 1^st^ branch order (ps < 0.0001). Ventrally, NRG1 did not affect number (p = 0.53; Fig. S9M) or density (p = 0.19; Fig. S9O) of synapses, although there were significant effects of branch order (ps < 0.0001), with lower numbers of puncta in the 1^st^ branch order versus 3^rd^ and 4^th^ order branches (ps = 0.0003), and highest density in the 1^st^ branch order versus subsequent three branch orders (ps ≤ 0.0003).

Somatic synapse-related puncta did not differ overall (p = 0.63; Fig. S9P) or in the dorsal (p = 0.89; Fig. S9Q) or ventral (p = 0.59; Fig. S9R) subregions; this was also the case for somatic synapse density (overall: p = 0.39; dorsal: p = 0.67; ventral: p = 0.23; Fig. 4E,F, S8S,T).

### Septotemporal characterization of cytogenesis

**Figure 5:**
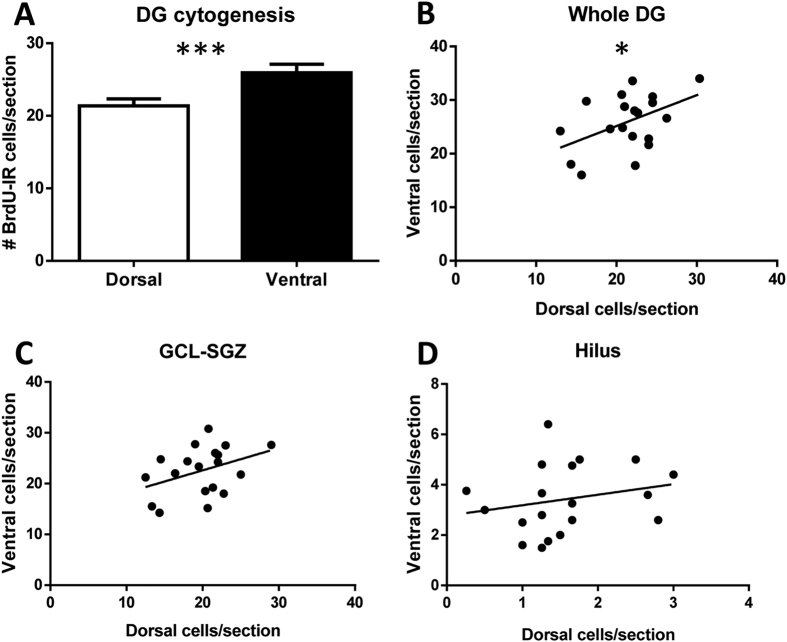
Septotemporal characterization of cytogenesis. (**A**) cytogenesis is higher in the ventral DG than in the dorsal DG. Cytogenesis in the subregions correlates significantly for the overall DG (**A**), and tends to correlate specifically in the granule cell layer and subgranular zone (GCL-SGZ) (**B**) but not the hilus (**C**). *p ≤ 0.05; **p ≤ 0.01; ***p ≤ 0.001.

With treatments pooled, cytogenesis was higher in the ventral DG overall (p = 0.0007; Fig. 5A), and in the SGZ-GCL (p = 0.028; Fig. S10A) and hilus (p < 0.0001; Fig. S10B) specifically. Overall DG cytogenesis was significantly correlated between the dorsal and ventral DG (r = 0.47; p = 0.041; Fig. 5B), and this tended to be the case specifically in the SGZ-GCL (r = 0.39; p = 0.099; Fig. 5C) but not the hilus (r = 0.23; p = 0.35; Fig. 5D).

### Septotemporal characterization of immature neuronal morphology

**Figure 6:**
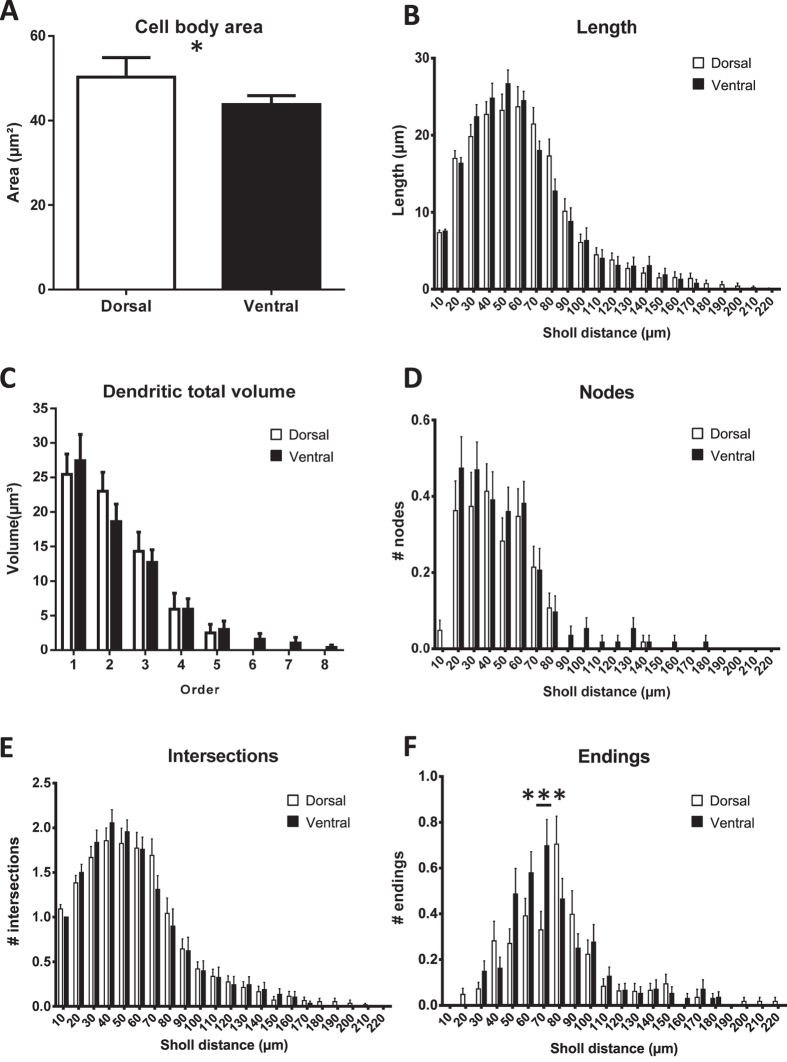
Septotemporal characterization of immature neuronal morphology. (**A**) cell body size was decreased for immature neurons in the ventral DG versus the dorsal DG. Dendritic length (**B**), volume (**C**), nodes (**D**), and intersections (**E**) did not differ for immature neurons between septotemporal subregions, although terminal dendritic endings were increased for ventral DG immature neurons (**F**) specifically 60–70 μm from the cell body.

Summarized characteristics of dorsal and ventral DG immature neurons are available in Table 2. With treatments pooled, cell body perimeter (p = 0.027; Fig. S10C) and area (p = 0.049; Fig. 6A) were smaller for immature neurons in the ventral DG. Feret max (p = 0.065; Fig. S10D), but not feret min (p = 0.13; Fig. S10E), tended to be larger in the dorsal DG. Cell body aspect ratio (p = 0.31; Fig. S10F), compactness (p = 0.27; Fig. S10G), convexity (p > 0.99; Fig. S10H), form factor (p = 0.30; Fig. S10I), roundness (p = 0.30; Fig. S10J), and solidity (p = 0.70; Fig. S10K) did not differ between subregions.

Overall, dendritic length (p = 0.89; Fig. S10L), volume (p = 0.77; Fig. S10M), surface area (p = 0.80; Fig. S10N), nodes (p = 0.49; Fig. S10O), endings (p = 0.51; Fig. S10P), and tortuosity (p = 0.95; Fig. S10Q) did not differ between subregions.

Dendritic length did not differ between dorsal and ventral subregions by branch order (p = 0.87; Fig. S10R) or sholl analysis (p = 0.85), although length was highest for 2^nd^ and 3^rd^ order branches (ps ≤ 0.0034) and between 10–80 μm from the cell body (ps < 0.0001; Fig. 6B). Mean dendritic length differed by branch order (p < 0.0001) and was highest for the first three branch orders versus subsequent orders (ps < 0.0001; Fig. S10S) but did not differ between subregions (p = 0.57).

Dendritic volume did not differ by subregion (p = 0.95), although volume differed by branch order (p < 0.0001) with volume highest in the first three branch orders (ps ≤ 0.0041; Fig. 6C). Dendritic mean volume did not differ by subregion (p = 0.93), although mean volume differed by branch order (p < 0.0001) and was higher in the first two branch orders than subsequent orders (ps ≤ 0.036; Fig. S10T).

Dendritic surface area did not differ by subregion (p = 0.73), although area was significantly higher in the first three branch orders (ps < 0.0001; Fig. S10U). Dendritic mean surface area did not differ by subregion (p = 0.64), but was significantly higher in the first three branch orders (ps ≤ 0.0020; Fig. S10V).

There was a tendency for number of branches to be highest in the ventral DG (p = 0.092), and they were highest in the 2^nd^ and 3^rd^ branch orders (ps ≤ 0.0001; Fig. S10W).

Number of nodes tended to be higher ventrally (p = 0.084), and were highest in the first three branch orders (ps ≤ 0.0001) and from 10–60 μm from the cell body (ps ≤ 0.013, except 40–50 vs 60–70: p = 0.48; Fig. 6D).

For dendritic intersections, dorsal and ventral subregions did not differ by Sholl analysis (p = 0.90), and intersections were more common 10–80 μm from the cell body than at other distances (ps ≤ 0.0061; Fig. 6E).

For dendritic endings, Sholl analysis revealed an interaction between subregion and Sholl distance (p = 0.0013); specifically, immature neurons in the ventral DG had significantly more endings 60–70 μm from the cell body (p = 0.0002; Fig. 6F).

### Septotemporal characterization of synapse-related immunoreactivity

**Figure 7:**
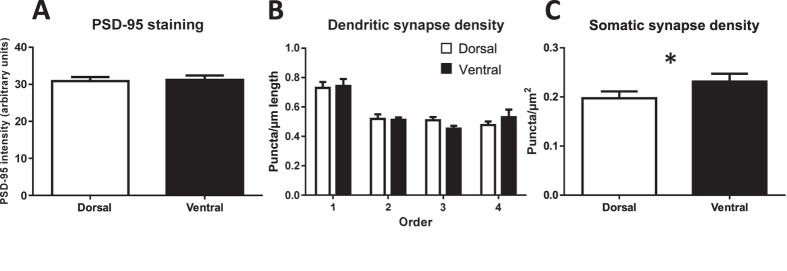
Septotemporal characterization of synapse-related immunoreactivity. (**A**) Synapse-related immunoreactivity in the molecular layer surrounding the DG does not differ between DG subregions along the septotemporal axis. (**B**) Dendritic synapse-related puncta density does not differ for immature neurons in the dorsal versus ventral DG; overall, density is highest in the first dendritic branch. (**C**) Somatic synapse-related puncta density is higher for immature neurons in the ventral DG. *p ≤ 0.05.

With treatments pooled, overall molecular layer synapse-related immunoreactivity did not differ between the dorsal and ventral DG (p = 0.84; Fig. 7A). However, there was heterogeneity in approximated synaptic density in specific zones around the molecular layer (Fig. 4B). Specifically, overall approximated synaptic density varied by zone (p < 0.0001), with the highest density in the suprapyramidal basal zone compared to the suprapyramidal apical (p = 0.0005), infrapyramidal apical (p < 0.0001), and infrapyramidal basal (p = 0.039) zones. Suprapyramidal apical density was higher than infrapyramidal apical density (p = 0.0004), and infrapyramidal basal density was higher than that of the infrapyramidal apical zone (p < 0.0001).

Notably, these distinctions are largely preserved across the septotemporal axis. In the dorsal DG, the highest density was in the suprapyramidal basal zone compared to the suprapyramidal apical (p = 0.0045), and infrapyramidal apical (p < 0.0001) zones, but not the infrapyramidal basal zone (p = 0.26; Fig. S9C). Infrapyramidal apical density was also lower than suprapyramidal apical (p = 0.011) and infrapyramidal basal density (p < 0.0001). Ventrally, the highest density was in the suprapyramidal basal zone compared to the suprapyramidal apical p = 0.0086), infrapyramidal apical (p < 0.0001), and infrapyramidal basal zones (p = 0.037; Fig. S9D). Infrapyramidal apical density was also lower than suprapyramidal apical (p = 0.0032) and infrapyramidal basal density (p = 0.0004).

Pooled overall number of dendritic synapse-related puncta per immature neuron did not differ between the dorsal and ventral DG (p > 0.99; Fig. S11A), and this was maintained when controlling for dendritic length (p = 0.71; Fig. S11B). Analyzing by branch order, there were no overall septotemporal differences for number (p = 0.81; Fig. S11C) or density (p = 0.97; Fig. 7B) of dendritic synapse-related puncta; however, there were significant main effects for branch order for synapse number and density (ps < 0.0001). Specifically, first-order synapse-related puncta were less abundant than second- (p = 0.0065), third- (p < 0.0001), and fourth-order synapse-related puncta (p < 0.0001), and second-order synapse-related puncta were less abundant than for third- (p = 0.0022) and fourth-order (p = 0.0068). Conversely, when correcting for branch-order length, first-order synapse-related density was higher than that of other branch-orders (ps < 0.0001).

Number of somatic synapse-related puncta did not differ by subregion (p = 0.22; Fig. S11D), but ventral DG immature neurons showed increased somatic synapse-related puncta density (p = 0.028; Fig. 7C).

## Discussion

Our results suggest that NRG1β does not affect intermediate stages of neurogenesis in immature adultborn DG neurons. The temporal resolution of the various studies examining the effects of NRG1 on hippocampal neurogenesis was obtained based on isolating cellular populations of particular age using BrdU labeling, focusing either shortly after division or four weeks hence^12^, or an intermediate amount of time in the current studies. Our findings indicate that the influence of NRG1 on hippocampal neurogenesis is temporally- and subregionally-specific, with effects only for proliferation in the ventral DG leading to neurogenic increases in this same subregion (as reported in ref. 12 and Table 1). Furthermore, this NRG1-mediated increase in neurogenesis is accompanied, four weeks after treatment cessation, by antidepressant-like effects^12^. Thus the current results further the characterization of the effects of NRG1 on hippocampal neurogenesis, initiated in our previous study^12^. We also found that NRG1 administration increased ErbB3 phosphorylation selectively in the ventral DG, corresponding to the location of increased proliferation of neuronal precursors (which express ErbB3) following NRG1 administration, and adding further support to findings indicating that NRG1 readily crosses the blood-brain barrier and affects brain processes^12^,^40^. This subregional specificity of ErbB3 phosphorylation and cell proliferation may be related to increased ErbB3 expression in neurogenic cells in the ventral DG compared to the dorsal DG^12^. The rapid timing of this effect is in line with previous research showing phosphorylation of ErbB receptors in the developing frontal cortex by peripherally-administered NRG1 within one hour of administration^41^. Downstream signaling for ErbB3 primarily involves PI3K/Akt, and ErbB3 has been shown to be required for proliferation of certain brain cells potentially through PI3K signaling, thus this signaling pathway might mechanistically link DG ErbB3 phosphorylation to the neurogenic cell proliferation effects we have observed^12^,^42^,^43^,^44^. Overall, the current results suggest that to the extent that changes in ventral DG neurogenesis underlie this change in emotion-related behavior four weeks after NRG1 administration (and ventral DG ErbB3 phosphorylation), it is changes to cell proliferation alone (and subsequent increases in immature functional neurons four weeks after proliferation), as opposed to additional contributions from other neurogenic stages, that contribute to this phenomenon.

The lack of effect of NRG1β administration on dendritogenesis or synaptogenesis may seem counter-intuitive, as previous *in vitro* studies examining cortical interneurons have reported increased dendritogenesis and synaptogenesis following NRG1β administration^45^,^46^. However, this appeared to be contingent upon ErbB4 expression^45^,^46^. Newborn granule cells in the adult DG lack ErbB4 but express ErbB3^12^, suggesting that NRG1-ErbB4 signaling can modulate dendritogenesis and synaptogenesis, whereas NRG1-ErbB3 signaling is involved in cell proliferation. Indeed, NRG1-ErbB3 signaling not only seems to stimulate the proliferation of neuronal precursors in the mature hippocampus^12^ but also stimulates proliferation in cancer cell lines^47^. In support of this, Ting *et al.*^46^ found that neither size nor number of synapses made by glutamatergic neurons, which have been shown to largely or completely lack ErbB4,^48^,^49^,^50^,^51^ were affected by NRG1β administration.

Comparison of the specificity of hippocampal neurogenic effects following NRG1 administration with other neurotrophic factors is partially confounded by incomplete characterization of these factors’ effects along the neurogenic timeline from proliferation to functionality. However, it has been shown that BDNF administration increases cell survival but not proliferation^21^, IGF administration increases proliferation and neuronal differentiation^23^, and VEGF increases proliferation but not survival^22^,^52^. These results highlight the potential specificity of neurogenic effects of neurotrophic factors, and underscore the importance of assessing at which particular neurogenic stages pro-neurogenic neurotrophic factors induce their effects.

To our knowledge, we also provide the first characterization of parameters that define and distinguish immature neurons in the dorsal and ventral DG. These include the novel identification of differences between these subregions in cell body size, somatic synapses, and dendritic branching. Although associating the differences observed between these particular immature neuronal populations with the distinct behavioral functions associated with either the dorsal or ventral hippocampus is beyond the scope of this study, these differences likely contribute to neuronal function. The smaller cell body size in ventral DG immature neurons is plausibly related to the previously-established maturational distinction between dorsal and ventral immature neurons, in which ventral DG immature neurons appear to mature more slowly with a wider window of excitability^13^,^53^,^54^. The observed increase in approximate somatic synapse density for ventral DG immature neurons is likely due to this reduced cell body size, as the total number of somatic synapse-related puncta per neuron did not differ by DG subregion. Interestingly, the increase in terminal dendritic branches in ventral DG immature neurons occurred 60–70 μm from the cell body, which roughly corresponds to the point at which developing dendrites emerge from the granule cell layer into the molecular layer, selectively receiving input from mossy cells^55^,^56^. This suggests that immature neurons in the ventral DG receive comparatively more input from neighboring mossy cells.

We find that cytogenesis, as determined by the number of BrdU-IR cells that proliferated prior to NRG1/saline administration and survived until the animal was sacrificed, was consistently higher in the ventral DG. Previous studies have found that, after water maze training, cell proliferation and neurogenesis were higher in the dorsal than ventral DG^57^,^58^. However, these studies examined density of neurogenic cells by volume, as opposed to the absolute number of cells as analyzed here, and thus this discrepancy may be explained by the volumetric difference between the dorsal and ventral DG. Consequently, it may be that the density of new cells is lower in the ventral DG, whereas overall cytogenesis as measured by the total number of adultborn cells is higher in this region, as reported here. This cytogenic heterogeneity along the septotemporal axis suggests that cytogenic assessments in which cell counts are averaged across the septotemporal axis may be less accurate than those assessing septotemporal subregions separately. However, we find that dorsal and ventral DG cytogenesis are significantly correlated, supporting the notion that a common set of factors regulates this dynamic phenomenon in both regions.

In addition, we identified heterogeneity in approximate dendritic synaptic density in the molecular layer that was maintained along the septotemporal axis; specifically, density was generally highest in the suprapyramidal basal zone and lowest in the infrapyramidal apical zone. To our knowledge, this is the first indication of such an unequal distribution in granule cell synapses in the molecular layer, and suggests that the resulting synaptic profile of adultborn neurons may be contingent on their precise position of integration within the granule cell layer.

We examined many parameters in order to characterize our morphological data as thoroughly as possible. Despite the number of analyses, NRG1 did not affect immature neuronal morphology, further supporting a lack of effect of NRG1 on cells of this neurogenic stage of development. The low number of specific dorsal-ventral statistically significant findings relative to the number of morphological comparisons argues against these findings arising due to inflated risk of type I statistical error. However, subsequent experimental validation of these results would determine this further.

Together, these results further characterize the effects of exogenous NRG1 administration on hippocampal neurogenesis across neurogenic stages. They indicate that the neurogenic influence of NRG1 is temporally and subregionally restricted to increases in ventral DG cell proliferation (likely through NRG1-ErbB3 activity) and resulting increases in the number of functional immature DG neurons. In addition, we also determined that the morphology and connectivity of immature neurons differs along the septotemporal axis of the DG, and hypothesize that the specificity of these subregional features may contribute to the functional differences that exist between the dorsal and ventral DG.

## Additional Information

**How to cite this article**: Mahar, I. *et al.* Effects of neuregulin-1 administration on neurogenesis in the adult mouse hippocampus, and characterization of immature neurons along the septotemporal axis. *Sci. Rep.* **6**, 30467; doi: 10.1038/srep30467 (2016).

## Supplementary Material

Supplementary Information

## Figures and Tables

**Table 1:** Specificity of effects of neuregulin-1 on adult hippocampal neurogenesis.

STAGE	OVERALL	DORSAL	VENTRAL
PROLIFERATION	↑	X	↑
DIFFERENTIATION	X	X	X
SURVIVAL	X	X	X
CELL BODY MORPHOLOGY	X	X	X
DENDRITIC MORPHOLOGY	X	X	X
SYNAPSE DEVELOPMENT	X	X	X
OVERALL NEUROGENESIS	↑	X	↑

Neuregulin-1 has temporally and subregionally specific neurogenic effects, wherein cell proliferation and overall neurogenesis are increased selectively in the ventral dentate gyrus following administration, and other neurogenic features (including neuronal differentiation, cell survival, cell body and dendritic morphology, and synapse formation) in immature neurons are unaffected. Proliferation, differentiation, and overall neurogenesis were assessed in ref. 12.

**Table 2:** Parameters of immature neurons in the dorsal and ventral dentate gyrus.

Immature neuronal parameters		
	Dorsal	Ventral
Cell body area	*50.28* ± *4.6 μm*	*43.81* ± *2.1 μm*
Dendritic length	191.5 ± 15.16 μm	182.7 ± 11.31 μm
Dendritic surface area	364.9 ± 35.39 μm^2^	338.5 ± 28.34 μm^2^
Dendritic volume	73.14 ± 7.89 μm^3^	69.27 ± 8.49 μm^3^
Dendritic nodes	2.31 ± 0.25	2.49 ± 0.23
Highest length	2^nd^ and 3^rd^ branch orders	2^nd^ and 3^rd^ branch orders
Nodes	Most prevalent 10–60 μm from the cell body	Most prevalent 10–60 μm from the cell body
Dendritic endings	*Fewer 60*–*70 μm from the cell body*	*More 60*–*70 μm from the cell body*
Dendritic synapses	Lowest (but most dense) in 1^st^ branch	Lowest (but most dense) in 1^st^ branch
Somatic synapse density	*Decreased versus ventral*	*Increased versus dorsal*
*Overall differentiating features*	*Larger cell body; decreased somatic synapse density; fewer terminal dendritic endings 60–70 μm from the cell body*	*Smaller cell body; increased somatic synapse density; more terminal dendritic endings 60–70 μm from the cell body*

Dendritic and synaptic synapses were assessed indirectly through PSD-95 immunolabeling. Statistically significant differences between immature neurons in the dorsal and ventreal dentate gyrus subregions are shown in italics.

## References

[b1] Kempermann G. & Gage F. H. Neurogenesis in the adult hippocampus. Novartis Foundation symposium 231, 220–235 (2000). 11131541

[b2] Deng W., Aimone J. B. & Gage F. H. New neurons and new memories: how does adult hippocampal neurogenesis affect learning and memory? Nature reviews. Neuroscience 11, 339–350, doi: 10.1038/nrn2822 (2010). 20354534 PMC2886712

[b3] Snyder J. S., Soumier A., Brewer M., Pickel J. & Cameron H. A. Adult hippocampal neurogenesis buffers stress responses and depressive behaviour. Nature 476, 458–461, doi: 10.1038/nature10287 (2011). 21814201 PMC3162077

[b4] Boldrini M. *et al.* Benzodiazepines and the potential trophic effect of antidepressants on dentate gyrus cells in mood disorders. Int J Neuropsychopharmacol 17, 1923–1933, doi: 10.1017/S1461145714000844 (2014). 24969726 PMC4374628

[b5] Boldrini M. *et al.* Hippocampal granule neuron number and dentate gyrus volume in antidepressant-treated and untreated major depression. Neuropsychopharmacology 38, 1068–1077, doi: 10.1038/npp.2013.5 (2013). 23303074 PMC3629406

[b6] Perera T. D. *et al.* Necessity of hippocampal neurogenesis for the therapeutic action of antidepressants in adult nonhuman primates. PloS one 6, e17600, doi: 10.1371/journal.pone.0017600 (2011). 21525974 PMC3078107

[b7] Malberg J. E., Eisch A. J., Nestler E. J. & Duman R. S. Chronic antidepressant treatment increases neurogenesis in adult rat hippocampus. J Neurosci 20, 9104–9110 (2000). 11124987 10.1523/JNEUROSCI.20-24-09104.2000 PMC6773038

[b8] Boldrini M. *et al.* Antidepressants increase neural progenitor cells in the human hippocampus. Neuropsychopharmacology 34, 2376–2389, doi: 10.1038/npp.2009.75 (2009). 19606083 PMC2743790

[b9] Ge S., Yang C. H., Hsu K. S., Ming G. L. & Song H. A critical period for enhanced synaptic plasticity in newly generated neurons of the adult brain. Neuron 54, 559–566, doi: 10.1016/j.neuron.2007.05.002 (2007). 17521569 PMC2040308

[b10] Aimone J. B., Deng W. & Gage F. H. Put them out to pasture? What are old granule cells good for, anyway…? Hippocampus 20, 1124–1125, doi: 10.1002/hipo.20867 (2010). 20848611

[b11] Fanselow M. S. & Dong H. W. Are the dorsal and ventral hippocampus functionally distinct structures? Neuron 65, 7–19, doi: 10.1016/j.neuron.2009.11.031 (2010). 20152109 PMC2822727

[b12] Mahar I. *et al.* Subchronic peripheral neuregulin-1 increases ventral hippocampal neurogenesis and induces antidepressant-like effects. PloS one 6, e26610, doi: 10.1371/journal.pone.0026610 (2011). 22028923 PMC3197569

[b13] Tanti A. *et al.* Region-dependent and stage-specific effects of stress, environmental enrichment, and antidepressant treatment on hippocampal neurogenesis. Hippocampus 23, 797–811, doi: 10.1002/hipo.22134 (2013). 23592526

[b14] Bannerman D. M. *et al.* Regional dissociations within the hippocampus–memory and anxiety. Neurosci Biobehav Rev 28, 273–283, doi: 10.1016/j.neubiorev.2004.03.004 (2004). 15225971

[b15] van Strien N. M., Cappaert N. L. & Witter M. P. The anatomy of memory: an interactive overview of the parahippocampal-hippocampal network. Nature reviews. Neuroscience 10, 272–282, doi: 10.1038/nrn2614 (2009). 19300446

[b16] Banasr M., Soumier A., Hery M., Mocaer E. & Daszuta A. Agomelatine, a new antidepressant, induces regional changes in hippocampal neurogenesis. Biol Psychiatry 59, 1087–1096, doi: 10.1016/j.biopsych.2005.11.025 (2006). 16499883

[b17] Elizalde N. *et al.* Sustained stress-induced changes in mice as a model for chronic depression. Psychopharmacology (Berl) 210, 393–406, doi: 10.1007/s00213-010-1835-6 (2010). 20401750

[b18] Lehmann M. L., Brachman R. A., Martinowich K., Schloesser R. J. & Herkenham M. Glucocorticoids orchestrate divergent effects on mood through adult neurogenesis. J Neurosci 33, 2961–2972, doi: 10.1523/JNEUROSCI.3878-12.2013 (2013). 23407954 PMC3711562

[b19] Mahar I., Bambico F. R., Mechawar N. & Nobrega J. N. Stress, serotonin, and hippocampal neurogenesis in relation to depression and antidepressant effects. Neurosci Biobehav Rev 38, 173–192, doi: 10.1016/j.neubiorev.2013.11.009 (2014). 24300695

[b20] Scharfman H. *et al.* Increased neurogenesis and the ectopic granule cells after intrahippocampal BDNF infusion in adult rats. Experimental neurology 192, 348–356, doi: 10.1016/j.expneurol.2004.11.016 (2005). 15755552

[b21] Schmidt H. D. & Duman R. S. Peripheral BDNF produces antidepressant-like effects in cellular and behavioral models. Neuropsychopharmacology 35, 2378–2391, doi: 10.1038/npp.2010.114 (2010). 20686454 PMC2955759

[b22] Jin K. *et al.* Vascular endothelial growth factor (VEGF) stimulates neurogenesis *in vitro* and *in vivo*. Proceedings of the National Academy of Sciences of the United States of America 99, 11946–11950, doi: 10.1073/pnas.182296499 (2002). 12181492 PMC129374

[b23] Aberg M. A., Aberg N. D., Hedbacker H., Oscarsson J. & Eriksson P. S. Peripheral infusion of IGF-I selectively induces neurogenesis in the adult rat hippocampus. J Neurosci 20, 2896–2903 (2000). 10751442 10.1523/JNEUROSCI.20-08-02896.2000 PMC6772218

[b24] Bertram I. *et al.* Immunohistochemical evidence for impaired neuregulin-1 signaling in the prefrontal cortex in schizophrenia and in unipolar depression. Ann N Y Acad Sci 1096, 147–156, doi: 10.1196/annals.1397.080 (2007). 17405926

[b25] Georgieva L. *et al.* Support for neuregulin 1 as a susceptibility gene for bipolar disorder and schizophrenia. Biol Psychiatry 64, 419–427, doi: 10.1016/j.biopsych.2008.03.025 (2008). 18466881

[b26] Stefansson H. *et al.* Neuregulin 1 and susceptibility to schizophrenia. American journal of human genetics 71, 877–892, doi: 10.1086/342734 (2002). 12145742 PMC378543

[b27] Marballi K., Cruz D., Thompson P. & Walss-Bass C. Differential neuregulin 1 cleavage in the prefrontal cortex and hippocampus in schizophrenia and bipolar disorder: preliminary findings. PloS one 7, e36431, doi: 10.1371/journal.pone.0036431 (2012). 22590542 PMC3349664

[b28] Rethelyi J. M. *et al.* Association study of NRG1, DTNBP1, RGS4, G72/G30, and PIP5K2A with schizophrenia and symptom severity in a Hungarian sample. American journal of medical genetics. Part B, Neuropsychiatric genetics: the official publication of the International Society of Psychiatric Genetics 153B, 792–801, doi: 10.1002/ajmg.b.31049 (2010). 19937977

[b29] Grossman A. W. *et al.* Developmental characteristics of dendritic spines in the dentate gyrus of Fmr1 knockout mice. Brain Res 1355, 221–227, doi: 10.1016/j.brainres.2010.07.090 (2010). 20682298 PMC3433497

[b30] Snyder J. S. *et al.* Adult-born hippocampal neurons are more numerous, faster maturing, and more involved in behavior in rats than in mice. J Neurosci 29, 14484–14495, doi: 10.1523/JNEUROSCI.1768-09.2009 (2009). 19923282 PMC2830901

[b31] Toni N. *et al.* Synapse formation on neurons born in the adult hippocampus. Nature neuroscience 10, 727–734, doi: 10.1038/nn1908 (2007). 17486101

[b32] Zhao C., Teng E. M., Summers R. G. Jr., Ming G. L. & Gage F. H. Distinct morphological stages of dentate granule neuron maturation in the adult mouse hippocampus. J Neurosci 26, 3–11, doi: 10.1523/JNEUROSCI.3648-05.2006 (2006). 16399667 PMC6674324

[b33] Hagihara H., Toyama K., Yamasaki N. & Miyakawa T. Dissection of hippocampal dentate gyrus from adult mouse. J Vis Exp, doi: 10.3791/1543 (2009). PMC3142893 19920804

[b34] Mignone J. L., Kukekov V., Chiang A. S., Steindler D. & Enikolopov G. Neural stem and progenitor cells in nestin-GFP transgenic mice. J Comp Neurol 469, 311–324, doi: 10.1002/cne.10964 (2004). 14730584

[b35] Brewer G. J. & Torricelli J. R. Isolation and culture of adult neurons and neurospheres. Nat Protoc 2, 1490–1498, doi: 10.1038/nprot.2007.207 (2007). 17545985

[b36] Noori H. R. & Fornal C. A. The appropriateness of unbiased optical fractionators to assess cell proliferation in the adult hippocampus. Front Neurosci 5, 140, doi: 10.3389/fnins.2011.00140 (2011). 22207833 PMC3245968

[b37] Sierksma A. S. *et al.* Chronic phosphodiesterase type 2 inhibition improves memory in the APPswe/PS1dE9 mouse model of Alzheimer’s disease. Neuropharmacology 64, 124–136, doi: 10.1016/j.neuropharm.2012.06.048 (2013). 22771768

[b38] Sierksma A. S. *et al.* Behavioral and neurobiological effects of prenatal stress exposure in male and female APPswe/PS1dE9 mice. Neurobiology of aging 34, 319–337, doi: 10.1016/j.neurobiolaging.2012.05.012 (2013). 22738723

[b39] Franklin K. & Paxinos G. The mouse brain in stereotaxic coordinates (Academic Press, 2007).

[b40] Kastin A. J., Akerstrom V. & Pan W. Neuregulin-1-beta1 enters brain and spinal cord by receptor-mediated transport. Journal of neurochemistry 88, 965–970 (2004). 14756818 10.1046/j.1471-4159.2003.02224.x

[b41] Abe Y., Namba H., Kato T., Iwakura Y. & Nawa H. Neuregulin-1 signals from the periphery regulate AMPA receptor sensitivity and expression in GABAergic interneurons in developing neocortex. J Neurosci 31, 5699–5709, doi: 10.1523/JNEUROSCI.3477-10.2011 (2011). 21490211 PMC6622838

[b42] Cook R. S. *et al.* ErbB3 ablation impairs PI3K/Akt-dependent mammary tumorigenesis. Cancer research 71, 3941–3951, doi: 10.1158/0008-5472.CAN-10-3775 (2011). 21482676 PMC3204389

[b43] Sathyamurthy A. *et al.* ERBB3-mediated regulation of Bergmann glia proliferation in cerebellar lamination. Development 142, 522–532, doi: 10.1242/dev.115931 (2015). 25564653 PMC4302995

[b44] Smirnova T. *et al.* Phosphoinositide 3-kinase signaling is critical for ErbB3-driven breast cancer cell motility and metastasis. Oncogene 31, 706–715, doi: 10.1038/onc.2011.275 (2012). 21725367 PMC3469325

[b45] Cahill M. E. *et al.* Control of interneuron dendritic growth through NRG1/erbB4-mediated kalirin-7 disinhibition. Mol Psychiatry 17, 1, 99–107, doi: 10.1038/mp.2011.35 (2012). 21483438 PMC3135693

[b46] Ting A. K. *et al.* Neuregulin 1 promotes excitatory synapse development and function in GABAergic interneurons. J Neurosci 31, 15–25, doi: 10.1523/JNEUROSCI.2538-10.2011 (2011). 21209185 PMC3078582

[b47] Andrique L. *et al.* ErbB3(80 kDa), a nuclear variant of the ErbB3 receptor, binds to the Cyclin D1 promoter to activate cell proliferation but is negatively controlled by p14ARF. Cellular signalling 24, 1074–1085, doi: 10.1016/j.cellsig.2012.01.002 (2012). 22261253

[b48] Bean J. C. *et al.* Genetic labeling reveals novel cellular targets of schizophrenia susceptibility gene: distribution of GABA and non-GABA ErbB4-positive cells in adult mouse brain. J Neurosci 34, 13549–13566, doi: 10.1523/JNEUROSCI.2021-14.2014 (2014). 25274830 PMC4180480

[b49] Chen Y. J. *et al.* ErbB4 in parvalbumin-positive interneurons is critical for neuregulin 1 regulation of long-term potentiation. Proceedings of the National Academy of Sciences of the United States of America 107, 21818–21823, doi: 10.1073/pnas.1010669107 (2010). 21106764 PMC3003111

[b50] Neddens J. *et al.* Conserved interneuron-specific ErbB4 expression in frontal cortex of rodents, monkeys, and humans: implications for schizophrenia. Biol Psychiatry 70, 636–645, doi: 10.1016/j.biopsych.2011.04.016 (2011). 21664604 PMC5040357

[b51] Vullhorst D. *et al.* Selective expression of ErbB4 in interneurons, but not pyramidal cells, of the rodent hippocampus. J Neurosci 29, 12255–12264, doi: 10.1523/JNEUROSCI.2454-09.2009 (2009). 19793984 PMC2774835

[b52] Fournier N. M., Lee B., Banasr M., Elsayed M. & Duman R. S. Vascular endothelial growth factor regulates adult hippocampal cell proliferation through MEK/ERK- and PI3K/Akt-dependent signaling. Neuropharmacology 63, 642–652, doi: 10.1016/j.neuropharm.2012.04.033 (2012). 22580375 PMC3392414

[b53] Piatti V. C. *et al.* The timing for neuronal maturation in the adult hippocampus is modulated by local network activity. J Neurosci 31, 7715–7728, doi: 10.1523/JNEUROSCI.1380-11.2011 (2011). 21613484 PMC3701257

[b54] Snyder J. S., Ferrante S. C. & Cameron H. A. Late maturation of adult-born neurons in the temporal dentate gyrus. PloS one 7, e48757, doi: 10.1371/journal.pone.0048757 (2012). 23144957 PMC3492442

[b55] Buckmaster P. S., Strowbridge B. W., Kunkel D. D., Schmiege D. L. & Schwartzkroin P. A. Mossy cell axonal projections to the dentate gyrus molecular layer in the rat hippocampal slice. Hippocampus 2, 349–362, doi: 10.1002/hipo.450020403 (1992). 1284975

[b56] Scheff S. W. & Price D. A. Synaptic density in the inner molecular layer of the hippocampal dentate gyrus in Alzheimer disease. Journal of neuropathology and experimental neurology 57, 1146–1153 (1998). 9862637 10.1097/00005072-199812000-00006

[b57] Snyder J. S., Glover L. R., Sanzone K. M., Kamhi J. F. & Cameron H. A. The effects of exercise and stress on the survival and maturation of adult-generated granule cells. Hippocampus 19, 898–906, doi: 10.1002/hipo.20552 (2009). 19156854 PMC2755652

[b58] Snyder J. S. *et al.* Septo-temporal gradients of neurogenesis and activity in 13-month-old rats. Neurobiology of aging 32, 1149–1156, doi: 10.1016/j.neurobiolaging.2009.05.022 (2011). 19632743 PMC2889161

