# Supplementary material for: Effects of neuregulin-1 administration on neurogenesis in the adult mouse hippocampus, and characterization of immature neurons along the septotemporal axis
Source: Sci Rep. 2016 Jul 29;6:30467. doi: 10.1038/srep30467 (PMC4965755; doi:10.1038/srep30467)
Supplement: Supplementary Information [file srep30467-s1.doc]

**Effects of neuregulin-1 administration on neurogenesis in the adult mouse hippocampus and characterization of immature neurons along the septotemporal axis**

Authors: Ian Mahar ^a,b^, Angus MacIsaac ^a^, John Junghan Kim ^a^, Calvin Qiang ^a^, Maria Antonietta Davoli ^a^, Gustavo Turecki ^a,b,c^, Naguib Mechawar ^a,b,c,*^

**^a^** McGill Group for Suicide Studies, Douglas Mental Health University Institute, 6875 LaSalle Blvd, Verdun, Québec, Canada H4H 1R3

**^b^** McGill University, Integrated Program in Neuroscience, Montreal Neurological Institute

^c^ McGill University, Department of Psychiatry

***Corresponding author**:

Naguib Mechawar ([naguib.mechawar@mcgill.ca](mailto:naguib.mechawar@mcgill.ca))

Douglas Mental Health University Institute

6875 LaSalle Blvd, Verdun, Québec, Canada H4H 1R3

Tel.: (514) 761-6131 (ext. 3365

**Supplemental materials:**


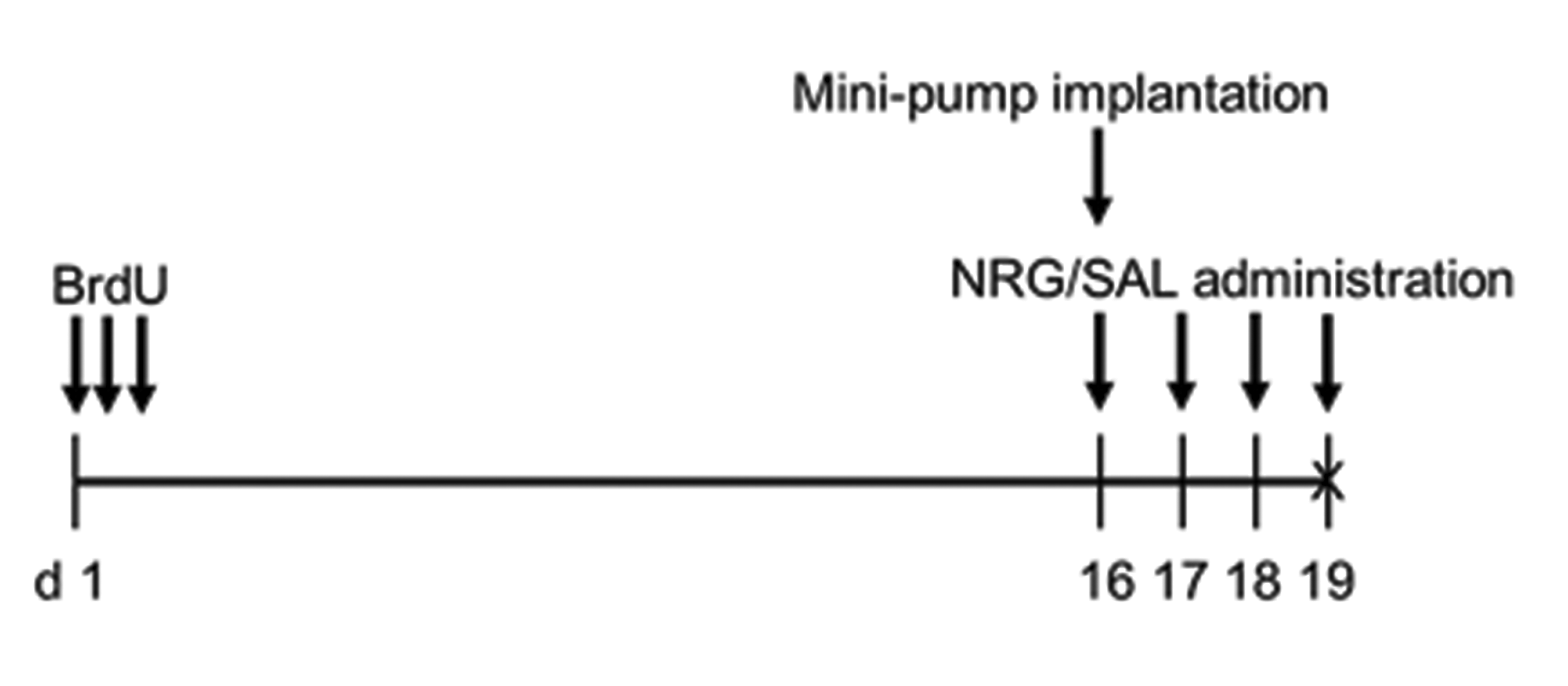


**Figure S1.** Experimental timeline. Mice were given three injections of BrdU on the first day of experimentation to label newborn cells. At day 16-19, corresponding to a period at which adultborn neurons undergo either apoptosis or morphological and synaptic development, neuregulin-1 (NRG1) or saline (SAL) was administered through subcutaneously implanted osmotic mini-pumps, and animals were sacrificed (X) following administration.



**Figure S2. A**: Widefield microscopy images of triple-labeling immunofluorescence (BrdU, blue; DCX, green; PSD-95, red), as used to initially identify BrdU-IR immature neurons prior to PSD-95 quantification (visualizing DCX and PSD-95) for these cells using an Apotome system. **B**, triple-labeling visualized with the Apotome system engaged. Scale bars: 10 µm.


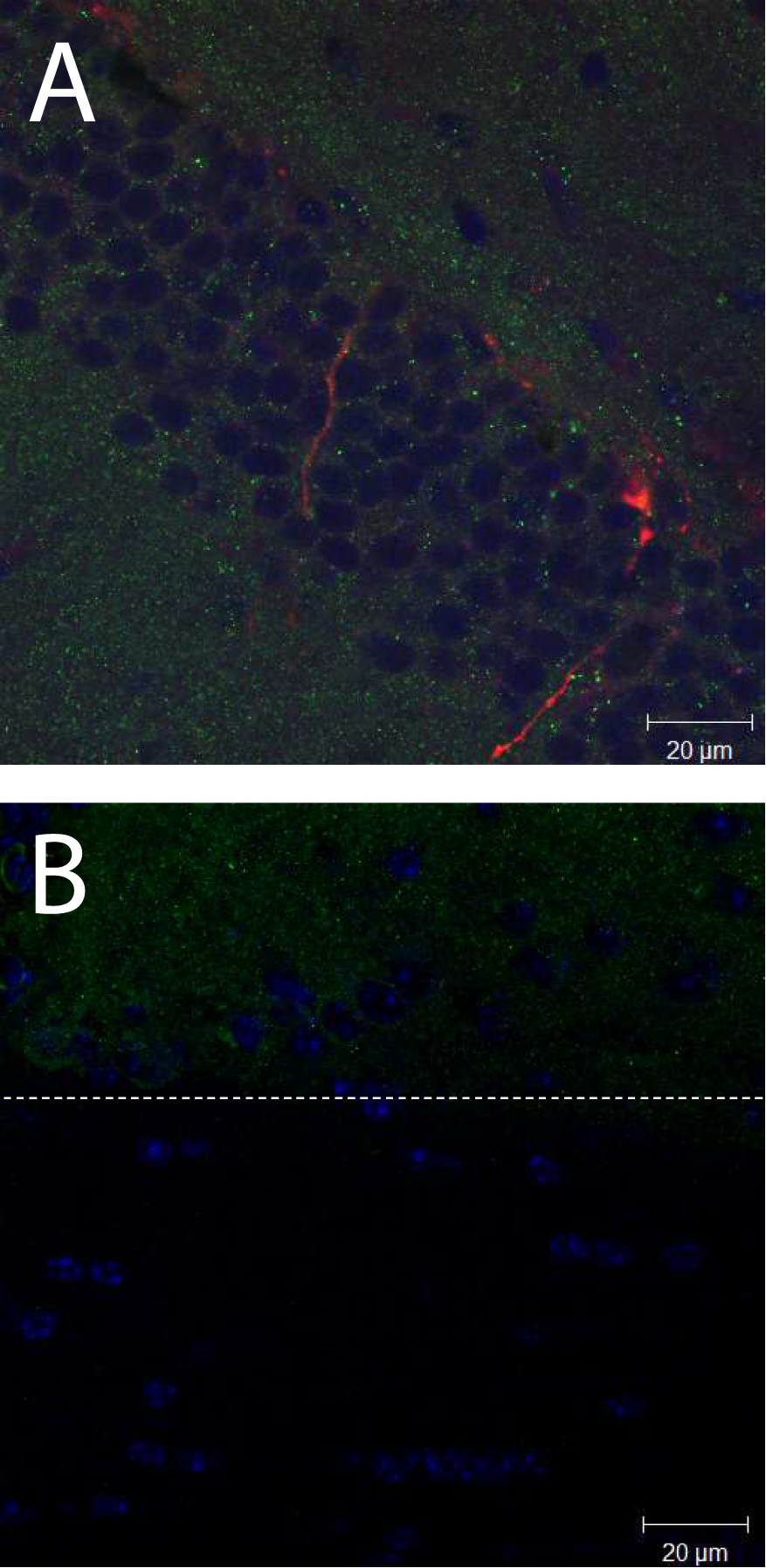
**Figure S3.** Comparison of PSD-95 staining in corpus callosum and hippocampal molecular layer. PSD-95 staining in the current protocol is absent from the corpus callosum but present in synaptically enriched regions; **A**, Dentate gyrus (DG); **B**, above dotted line (approximated): cortex; below: corpus callosum. Red (**A**), DCX; blue, DAPI; green, PSD-95. Scale bars: 20 µm

**
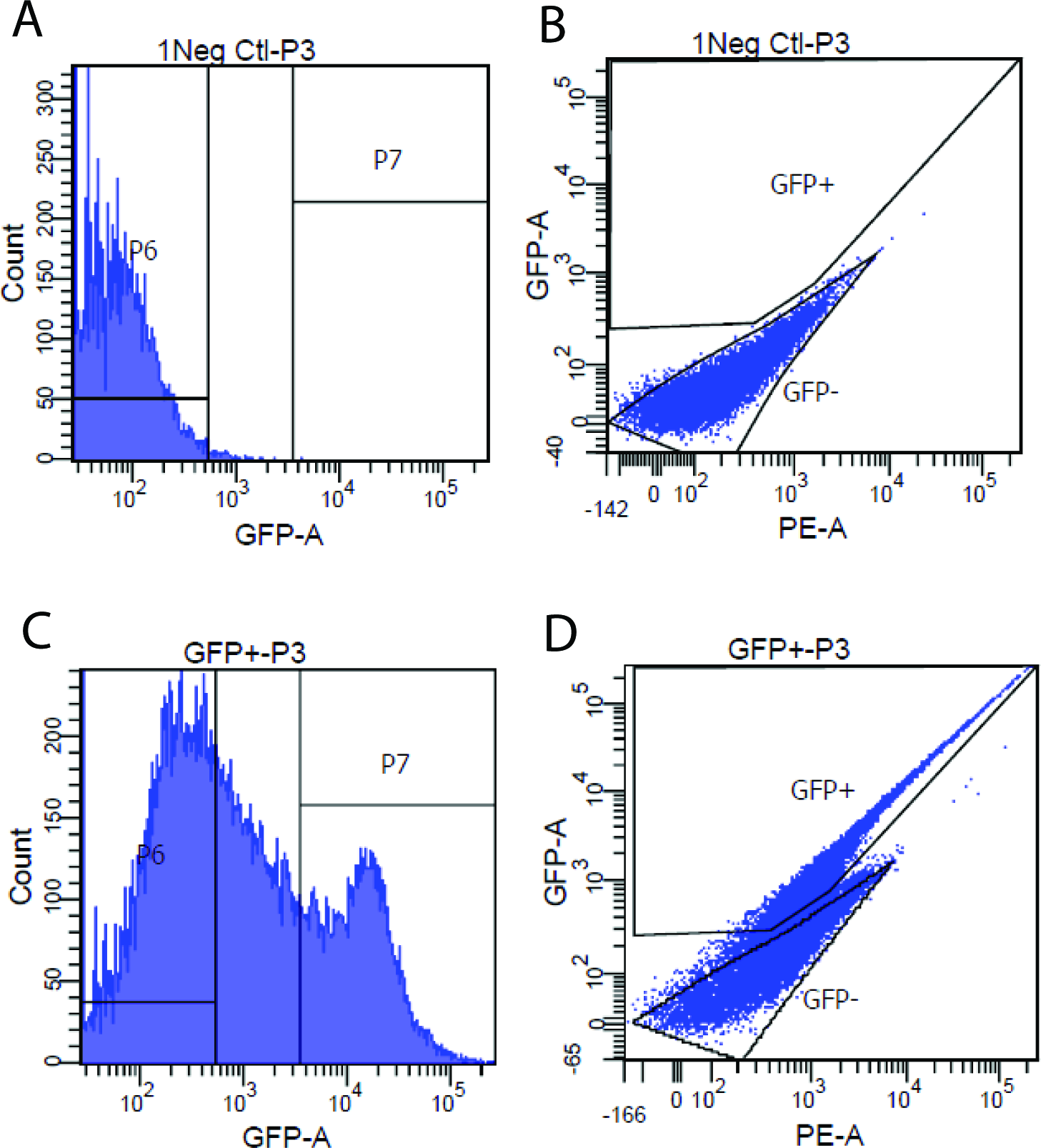
**

**Figure S4**. Fluorescence-assisted cell sorting confirmation of nestin-green fluorescent protein (GFP) cells in dissected DG, in order to verify dissection anatomical accuracy. **A**, sample histogram of sorted cell population in a dissection from a nestin-GFP-negative (non-transgenic) mouse, revealing a homogenous low-fluorescence cellular population. **B**, GFP signal intensity dot plot relative to phycoerythrin (PE) autofluorescence from a dissection from a non-transgenic mouse, revealing an absence of GFP+ fluorescence. **C**, sample histogram of a sorted cell population in a dissection from a nestin-GFP+ mouse, revealing a high GFP fluorescence signal and two distinct cellular populations. **D**, GFP signal intensity dot plot of a sorted cell population in a dissection from a nestin-GFP+ mouse, revealing a distinct GFP+ cell population that clusters at higher fluorescent signal intensity.


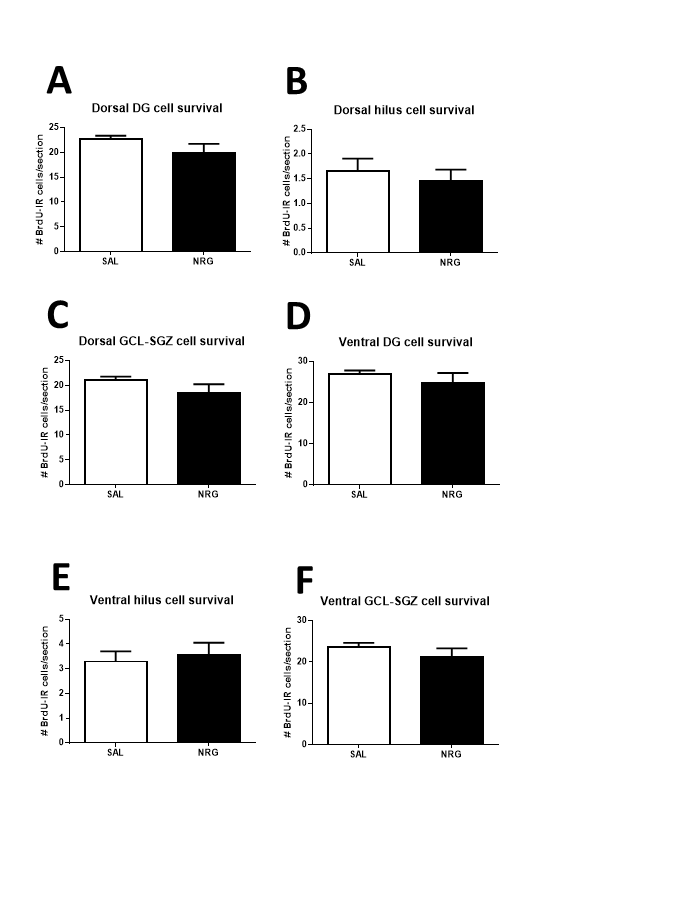


**Figure S5.** Additional findings on effects of neuregulin-1 administration on cell survival. NRG1 administration did not affect cell survival in the dorsal (**A**) or ventral (**B**) overall DG, or specifically in the subgranular zone and granular cell layer dorsally (**C**) or ventrally (**D**) or in the hilus dorsally (**E**) or ventrally (**F**).


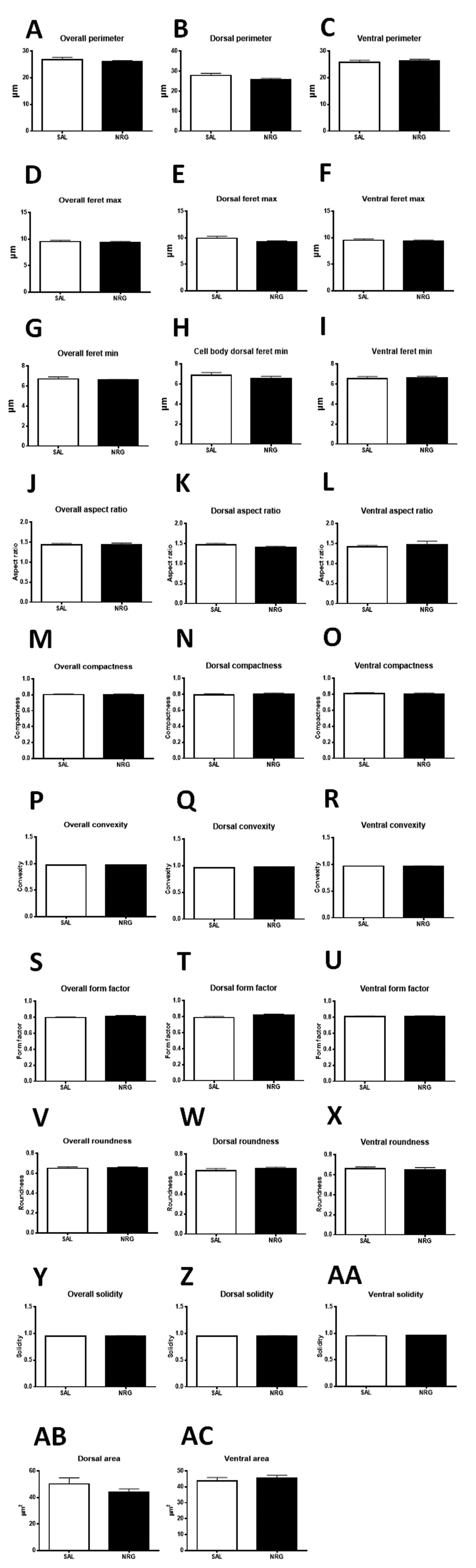


**
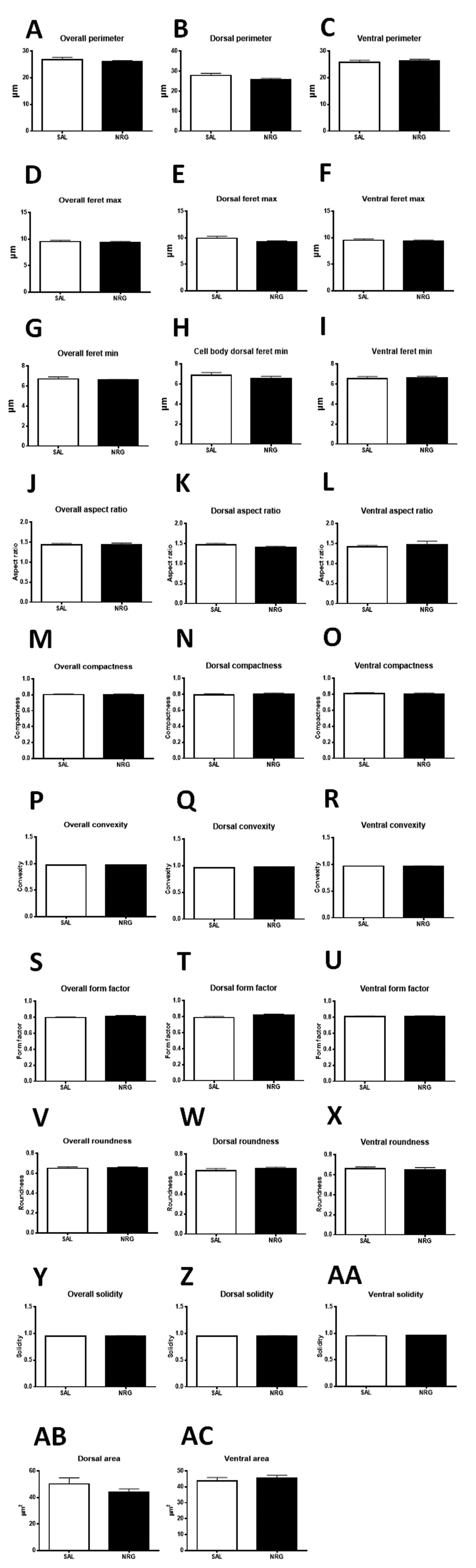
**

**Figure S6.** Additional findings on effects of neuregulin-1 administration on cell body morphological development. NRG1 administration did affect overall, dorsal, or ventral immature neuronal cell body perimeter (**A-C**), feret max (**D-F**), feret min (**G-I**), aspect ratio (**J-L**), compactness (**M-O**), convexity (**P-R**), form factor (**S-U**), roundness (**V-X**), solidity (**Y-AA**), or area (**AB-AC; Fig. 3C**).


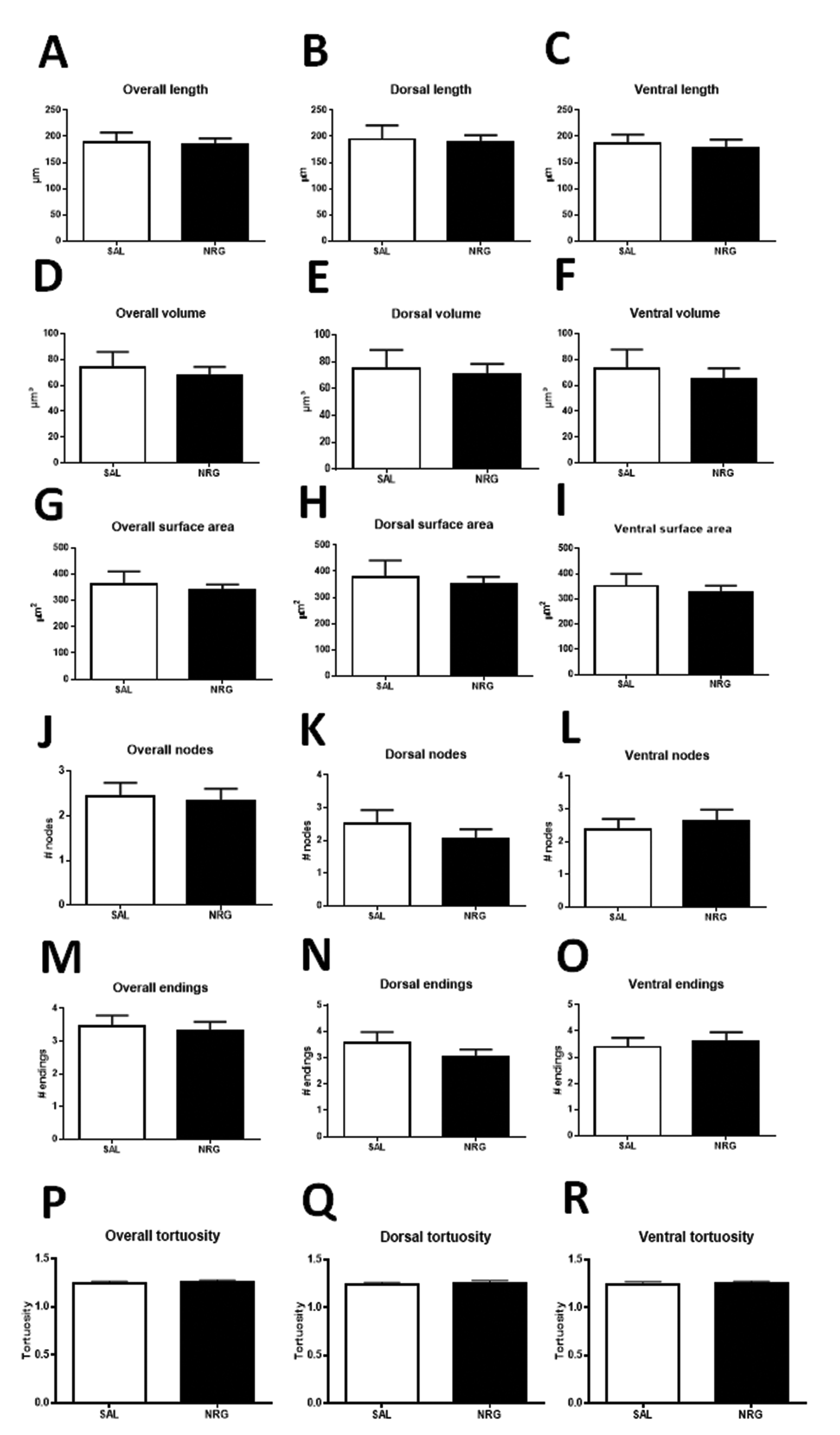


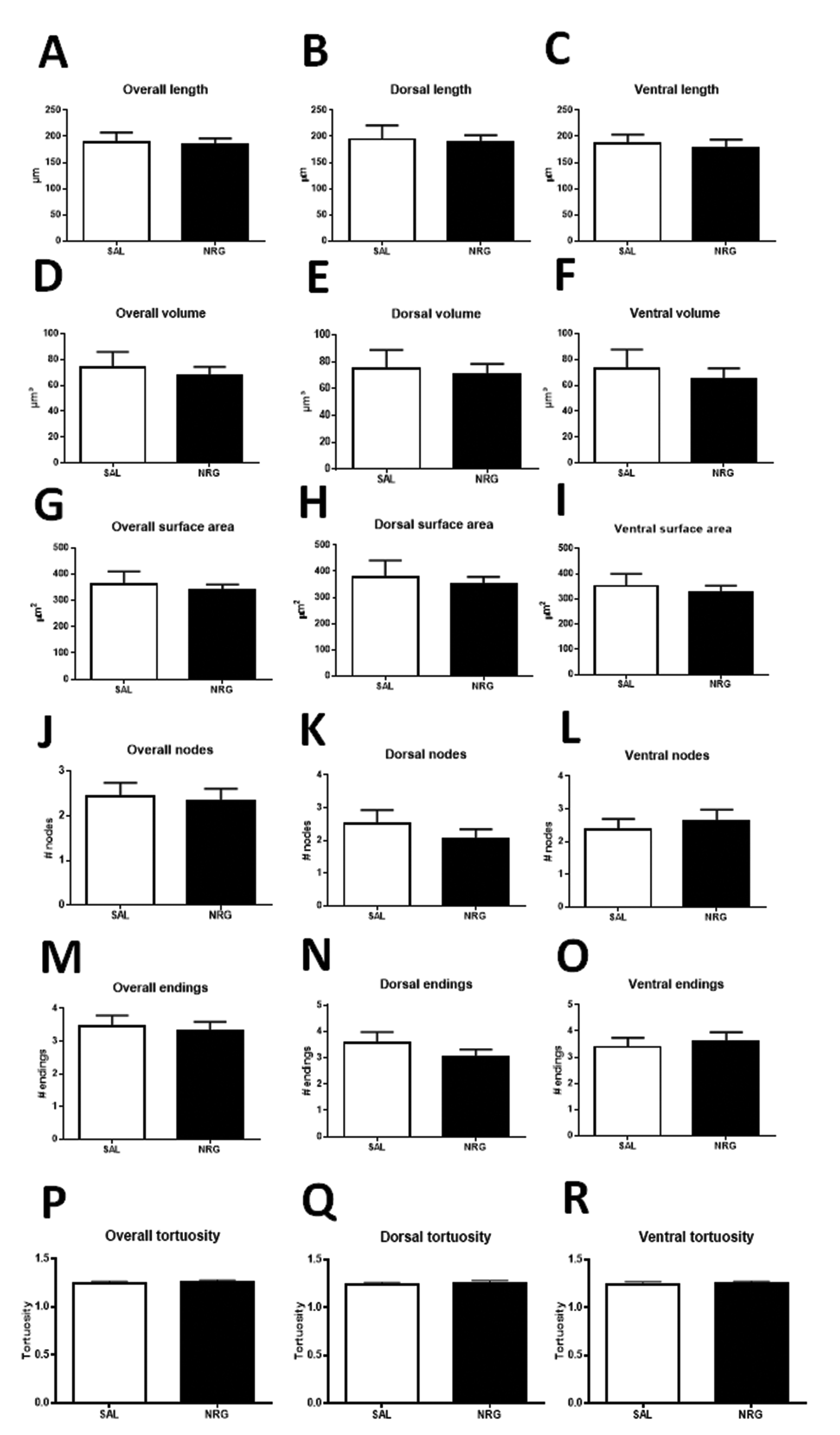


**Figure S7.** Additional findings on effects of neuregulin-1 administration on total dendritic morphological development. NRG1 administration did affect overall, dorsal, or ventral immature neuronal dendritic length (**A-C**), volume (**D-F**), surface area (**G-I**), nodes (**J-L**), ends (**M-O**), or tortuosity (**P-R**).


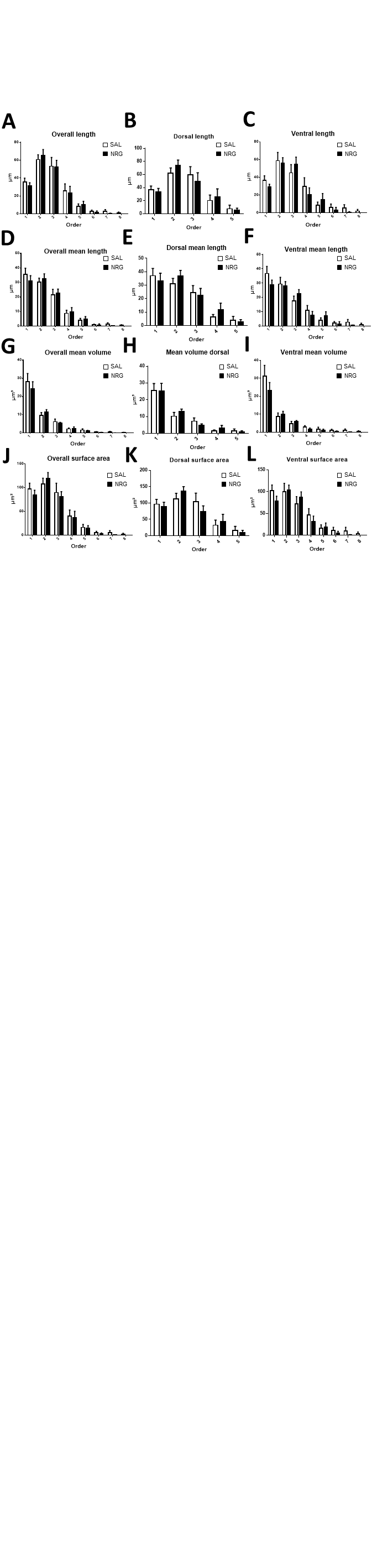


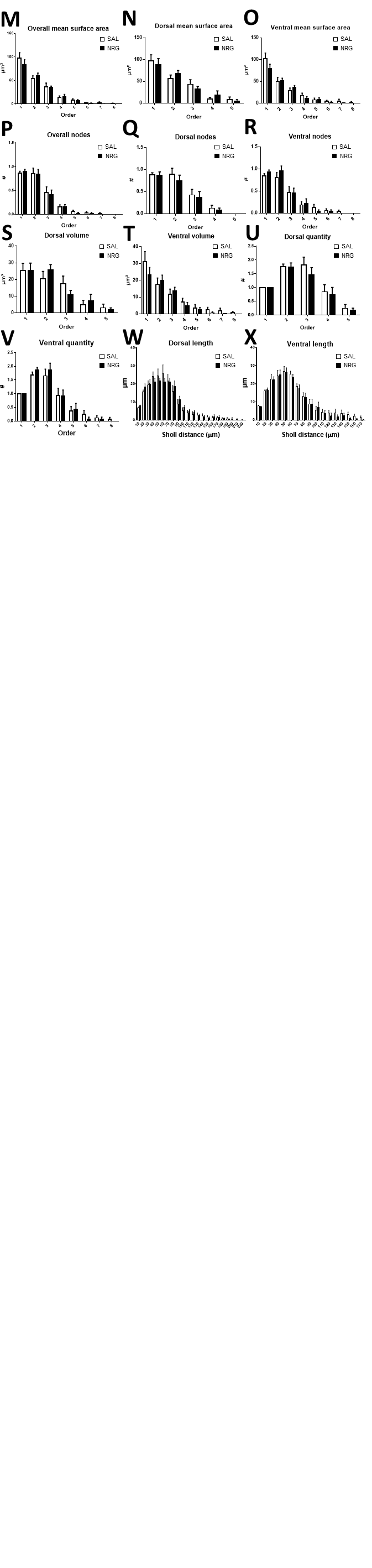


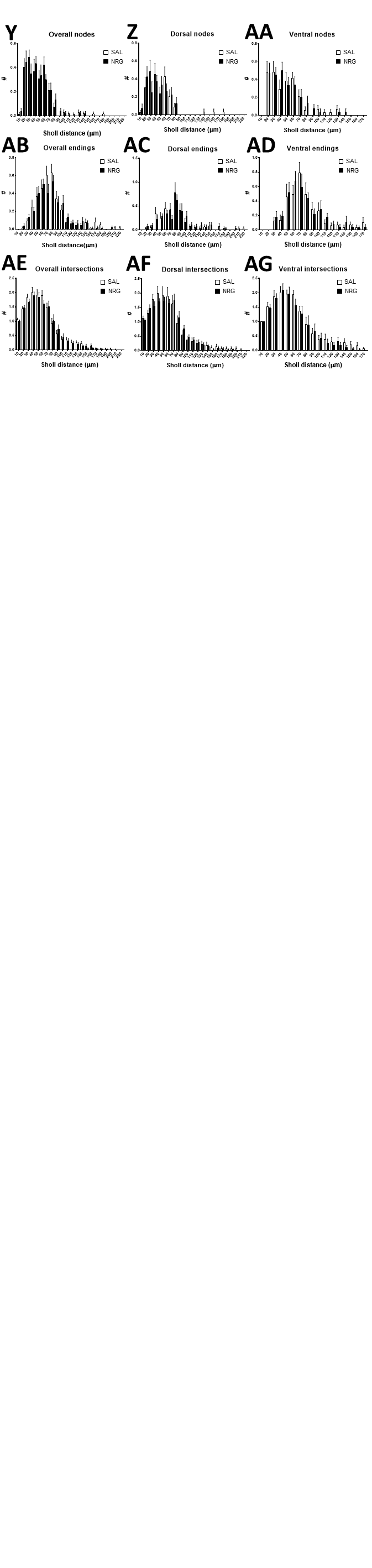


**Figure S8.** Additional findings on effects of neuregulin-1 administration on dendritic morphological development by branch order and Sholl analyses. By branch order, NRG1 administration did affect overall, dorsal, or ventral immature neuronal dendritic length (A-C), mean length (**D-F**), mean volume (**G-I**), surface area (**J-L**), mean surface area (**M-O**), nodes (**P-R**), ends (**M-O**), volume (**S-T; Fig. 3E**), or quantity (U-W). By Sholl analysis, NRG1 administration did not affect dendritic length (**W-X; Fig. 3D**), nodes (**Y-AA**), endings (**AB-AD**), or intersections (**AE-AG**).


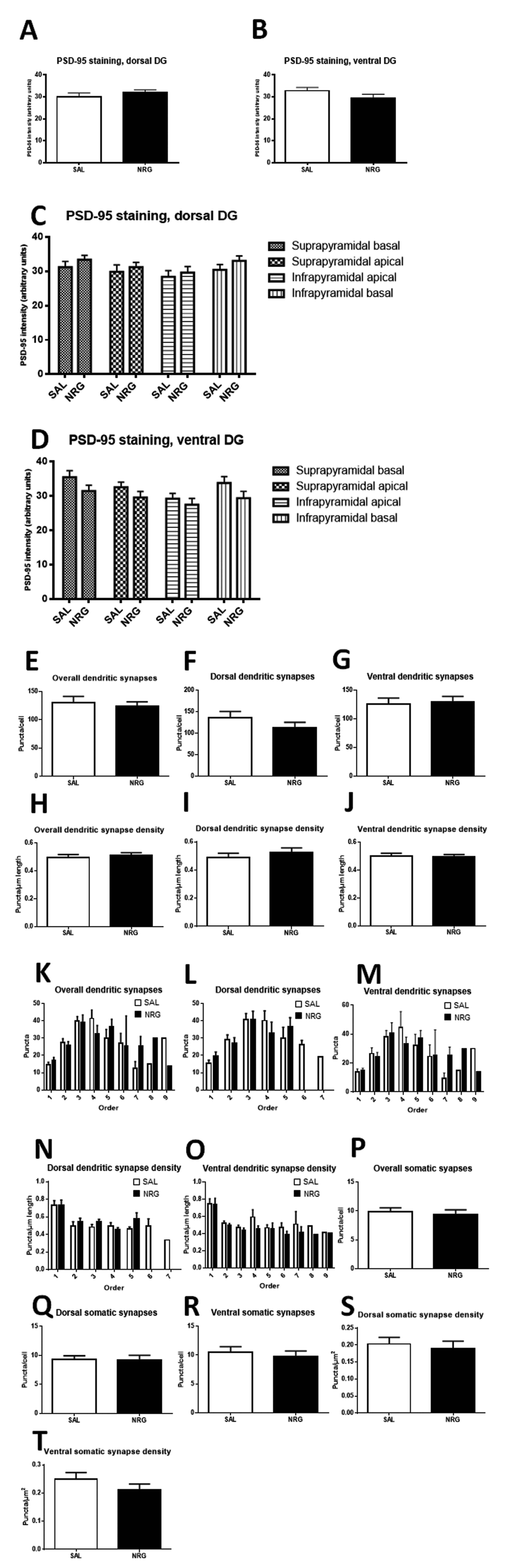


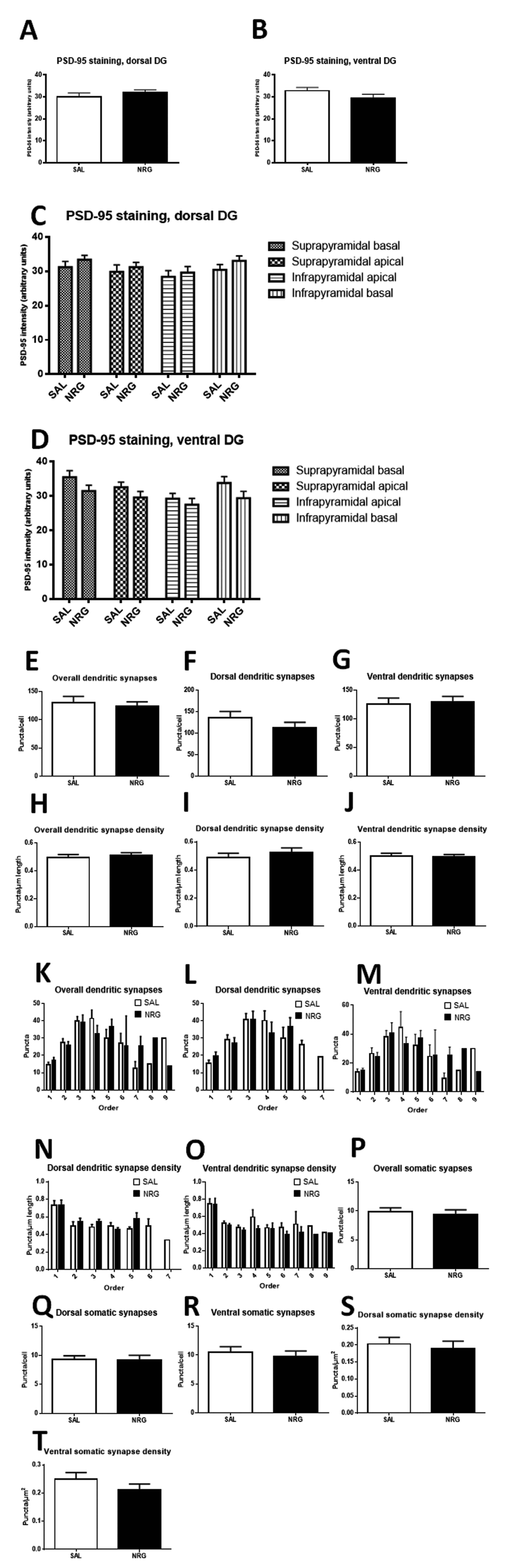


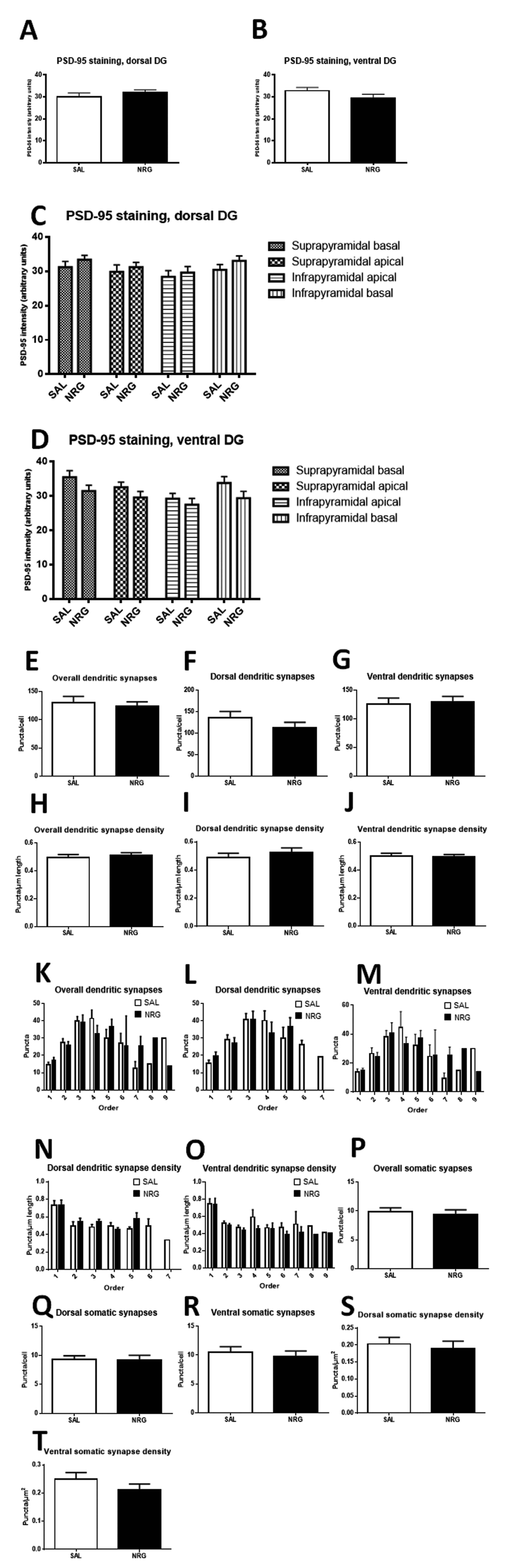


**Figure S9.** Additional findings on effects of neuregulin-1 administration on synapse development, as approximated by PSD-95 immunostaining. NRG1 did not affect total molecular layer synapse-related immunoreactivity density in the dorsal or ventral subregions, overall (**A-B**) or analyzed by molecular layer zone (**C-D**). Examining immature neurons specifically, NRG1 did not affect total dendritic synapse-related puncta numbers (**E-G**) or density (**H-J**), and this was maintained when examining by dendritic branch order (**K-O; Fig. 4D**). Number (**P-R**) and density (**S-T, Fig. 4F**) of somatic synapse-related puncta was unaffected by treatment.


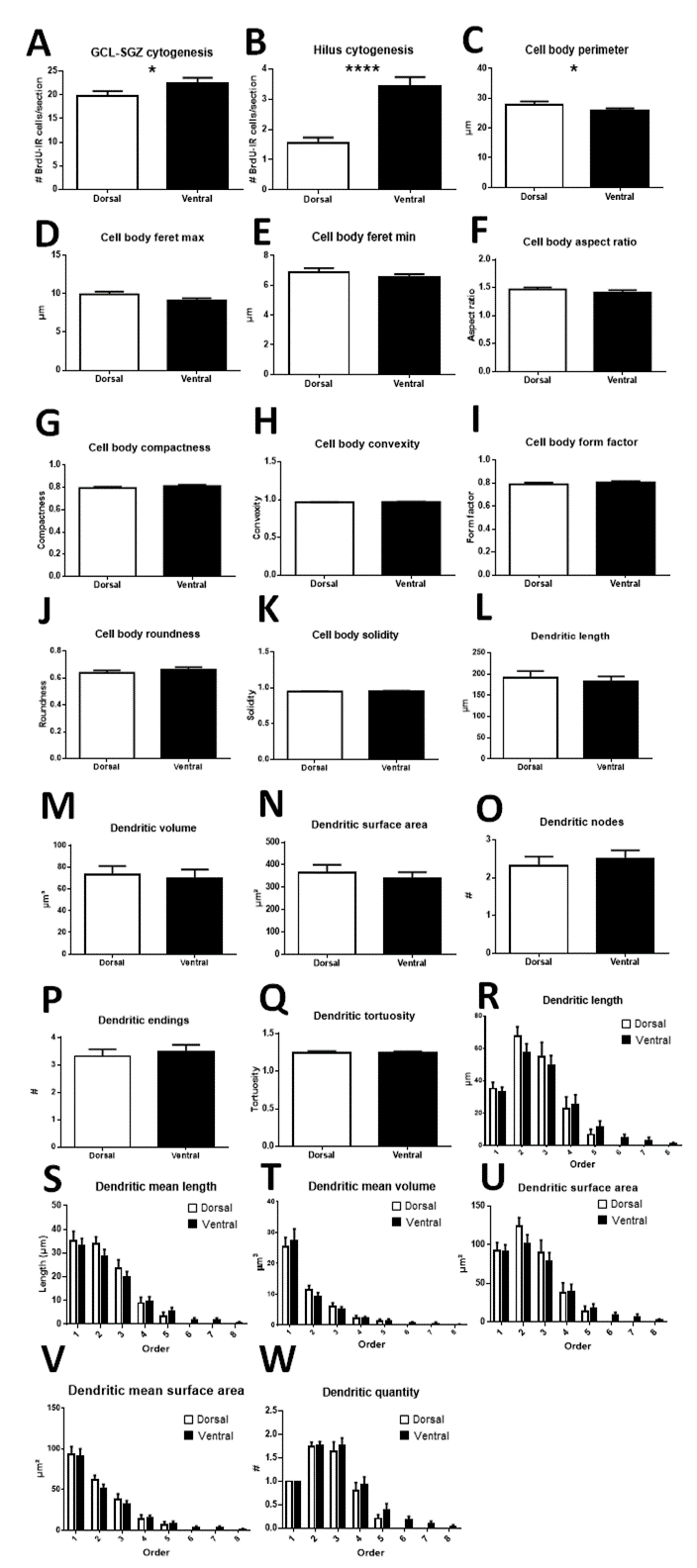


**
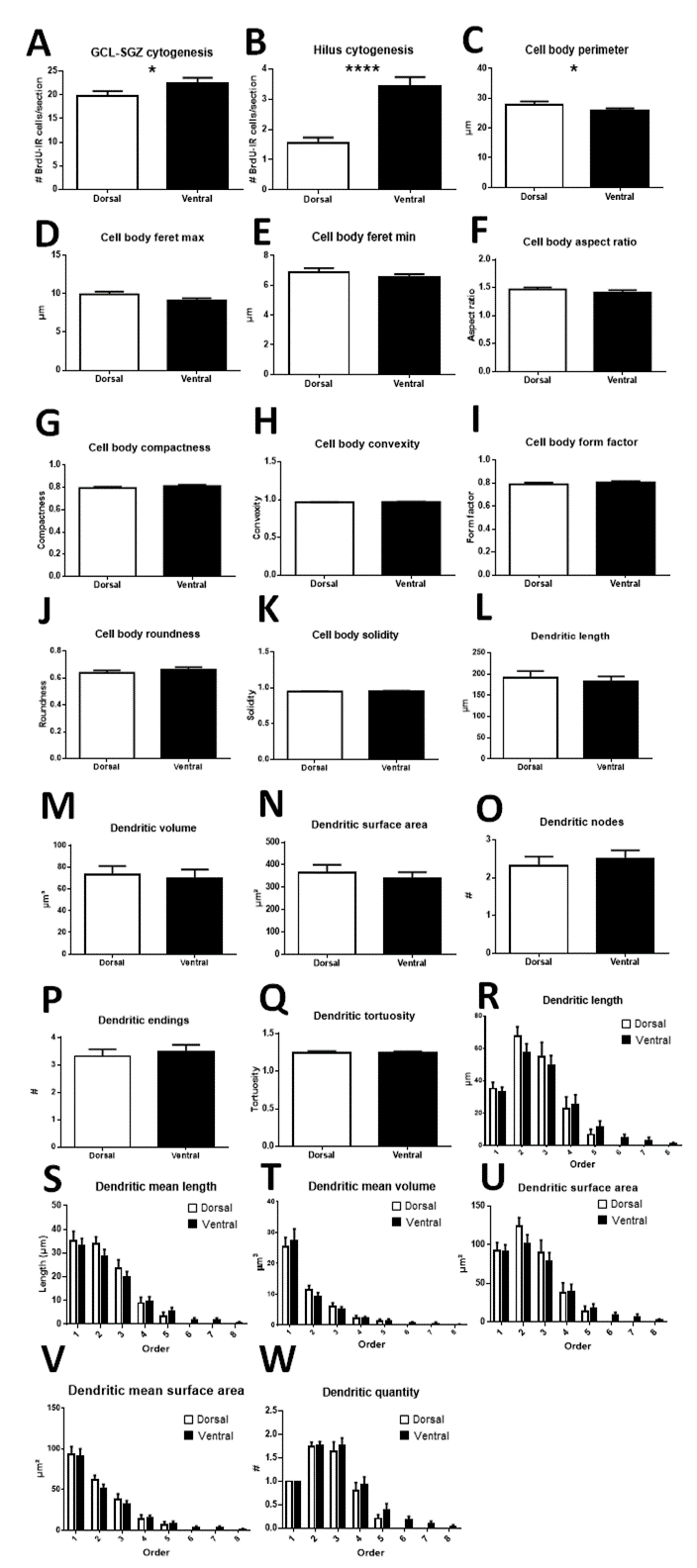
**

**Figure S10.** Additional findings on septotemporal characterization of cytogenesis and immature neuronal morphology. Cytogenesis was higher in the ventral granule cell layer and subgranular zone (**A**) and hilus (**B**) compared to dorsally. **C**, cell body perimeter was lower in immature neurons in the ventral versus dorsal DG. Dorsal and ventral immature neuronal cell body feret max (**D**), feret min (**E**), aspect ratio (**F**), compactness (**G**), convexity (**H**), form factor (**I**), roundness (**J**), or solidity (**K**) did not differ between groups. Immature neurons did not differ by subregion for total dendritic length (**L**), volume (**M**), surface area (**N**), nodes (**O**), ends (**P**), or tortuosity (**Q**). Analyzing by branch order, there were no septotemporal differences in dendritic length (**R**), mean dendritic length (**S**), mean volume (**T**), dendritic surface area (**U**) or mean surface area (**V**), or dendritic quantity (**W**). *, p≤0.05; **, p≤0.01; ***, p≤0.001; ****, p≤0.0001.


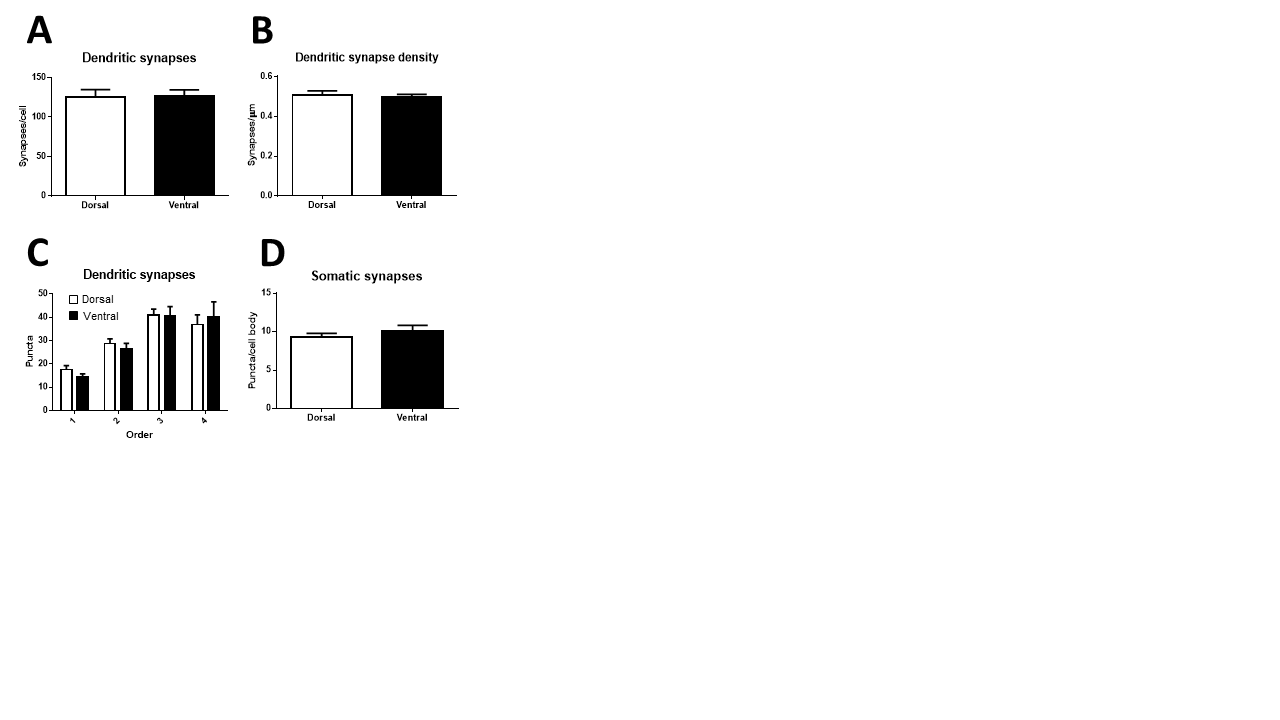


**Figure S11.** Additional findings on septotemporal characterization of synaptic development. Number (**A**) and density (**B**) of total dendritic synapse-related puncta did not differ between the dorsal and ventral DG. **C**, Number of synapse-related puncta did not differ between subregions by branch analysis. **D**, total number of somatic synapse-related puncta did not differ between subregions.
